# Mortality Among Young Adults Born Preterm and Early Term in 4 Nordic Nations

**DOI:** 10.1001/jamanetworkopen.2020.32779

**Published:** 2021-01-08

**Authors:** Kari Risnes, Josephine Funck Bilsteen, Paul Brown, Anna Pulakka, Anne-Marie Nybo Andersen, Signe Opdahl, Eero Kajantie, Sven Sandin

**Affiliations:** 1 Department of Clinical and Molecular Medicine, Faculty of Medicine and Health Science, Norwegian University of Science and Technology, Trondheim, Norway; 2 Department of Research, Innovation, and Education, Children’s Clinic, St Olavs Hospital, Trondheim University Hospital, Trondheim, Norway; 3 Department of Paediatrics, Hvidovre University Hospital, Hvidovre, Denmark; 4 Section of Epidemiology, Department of Public Health, University of Copenhagen, Copenhagen, Denmark; 5 Department of Public Health Solutions, Finnish Institute for Health and Welfare, Helsinki and Oulu, Finland; 6 Department of Public Health and Nursing, Faculty of Medicine and Health Science, Norwegian University of Science and Technology, Trondheim, Norway; 7 Research Unit for Pediatrics, Pediatric Neurology, Pediatric Surgery, Child Psychiatry, Dermatology, Clinical Genetics, Obstetrics and Gynecology, Otorhinolaryngology, and Ophthalmology, Medical Research Center Oulu, Oulu University Hospital and University of Oulu, Oulu, Finland; 8 Children’s Hospital, Helsinki University Hospital, University of Helsinki, Helsinki, Finland; 9 Department of Medical Epidemiology and Biostatistics, Karolinska Institutet, Stockholm, Sweden; 10 Jockey Club School of Public Health and Primary Care, The Chinese University of Hong Kong, Hong Kong Special Administrative Region; 11 Department of Psychiatry, Icahn School of Medicine at Mount Sinai, New York, New York; 12 Seaver Autism Center for Research and Treatment at Mount Sinai, New York, New York

## Abstract

**Importance:**

Adverse long-term outcomes in individuals born before full gestation are not confined to individuals born at extreme gestational ages. Little is known regarding mortality patterns among individuals born in the weeks close to ideal gestation, and the exact causes are not well understood; both of these are crucial for public health, with the potential for modification of risk.

**Objective:**

To examine the risk of all-cause and noncommunicable diseases (NCD) deaths among young adults born preterm and early term.

**Design, Setting, and Participants:**

This multinational population-based cohort study used nationwide birth cohorts from Norway, Sweden, Denmark, and Finland for individuals born between 1967 and 2002. Individuals identified at birth who had not died or emigrated were followed up for mortality from age 15 years to 2017. Analyses were performed from June 2019 to May 2020.

**Exposures:**

Categories of gestational age (ie, moderate preterm birth and earlier [23-33 weeks], late preterm [34-36 weeks], early term [37-38 weeks], full term [39-41 weeks] and post term [42-44 weeks]).

**Main Outcomes and Measures:**

All-cause mortality and cause-specific mortality from NCD, defined as cancer, diabetes, chronic lung disease, and cardiovascular disease (CVD).

**Results:**

A total of 6 263 286 individuals were followed up for mortality from age 15 years. Overall, 339 403 (5.4%) were born preterm, and 3 049 100 (48.7%) were women. Compared with full-term birth, the adjusted hazard ratios (aHRs) for all-cause mortality were 1.44 (95% CI, 1.34-1.55) for moderate preterm birth and earlier; 1.23 (95% CI, 1.18-1.29) for late preterm birth; and 1.12 (95% CI, 1.09-1.15) for early-term birth. The association between gestational age and all-cause mortality were stronger in women than in men (*P* for interaction = .03). Preterm birth was associated with 2-fold increased risks of death from CVD (aHR, 1.89; 95% CI, 1.45-2.47), diabetes (aHR, 1.98; 95% CI, 1.44-2.73), and chronic lung disease (aHR, 2.28; 95% CI, 1.36-3.82). The main associations were replicated across countries and could not be explained by familial or individual confounding factors.

**Conclusions and Relevance:**

The findings of this study strengthen the evidence of increased risk of death from NCDs in young adults born preterm. Importantly, the increased death risk was found across gestational ages up to the ideal term date and includes the much larger group with early-term birth. Excess mortality associated with shorter gestational age was most pronounced for CVDs, chronic lung disease, and diabetes.

## Key Points

**Question:**

Are all-cause and noncommunicable disease (NCD) mortality risks higher amomg adults born preterm and early term compared with those born at full term?

**Findings:**

This cohort study of more than 6.1 million individuals from 4 Nordic birth cohorts provided found all degrees of preterm birth and early-term birth are associated with increased risk of premature adult death. Excess mortality associated with shorter gestation was pronounced for death from NCDs, such as cardiovascular diseases, chronic lung disease, and diabetes.

**Meaning:**

These findings suggest that increased early adult mortality associated with shorter gestation includes excess risk in the large group of adults born late preterm and early term and is pronounced for NCD deaths.

## Teaser

This cohort study examines the risk of all-cause and noncommunicable disease deaths among young adults born preterm and early term in 4 Nordic countries.

## Introduction

Globally, 15 million pregnancies each year, 1 in 10, result in preterm birth, ie, birth before 37 weeks of gestation.^1,2^ Improved survival after preterm birth is among the most striking advances of modern health care, and in recent birth cohorts, more than 90% of those born preterm reach adulthood.^2^ Their lifelong health is of great interest not only to these individuals and their families but also to health care systems and society. So far, studies investigating long-term health and disease after preterm birth have mostly been limited to early adulthood among those with the highest risk, namely those born extremely (<28 weeks) and very (28-32 weeks) preterm.^3,4,5^ However, recent reports suggest that adverse long-term outcomes are not confined to extreme gestational ages, considering that children born just a few weeks before term or even early term had higher risk.^6^

The notion that early life is a vulnerable period, when even subtle disruptions in the development of organ systems may lead to adverse health outcomes in adulthood, is far from new.^7,8^ While many studies that have followed up individuals born in the mid to late 20th century demonstrate robust associations between lower birth weight and higher cardiometabolic disease risk and mortality,^9,10^ only some could assess preterm birth as a factor associated with these outcomes. Furthermore, results are mixed, and their generalizability to individuals entering adulthood today may be limited.^9,11,12^ A growing body of evidence from clinical follow-up studies associates shorter gestation with higher levels of risk factors for noncommunicable diseases (NCDs).^13,14,15^ For cancer, studies on associations with gestational age are few,^16^ while larger birth size has been associated with higher adult cancer risk.^10,17^

In most clinical follow-up studies of individuals born preterm, the participants are still too young for meaningful estimation of NCD risk and premature adult death. However, the nationwide medical birth registries established in the Nordic countries in the 1960s and 1970s enable follow-up into adulthood for large groups of individuals born preterm. Reports on gestational age and mortality in the Swedish population registries^18,19,20^ suggest that lower gestational age is associated with increased mortality in young adulthood. Norwegian data indicate similar patterns.^21^ The authors of the previous reports on Swedish data recently reviewed the evidence for preterm birth and adult mortality^22^ and found that studies from Sweden, Norway, and Australia had reported an association between preterm birth and adult mortality but that heterogeneity in analyses and reporting did not allow aggregation or any in-depth comparison of results.

Here, in what is, to our knowledge, the largest study to date and with a maximal follow-up until age 50 years, we expand previous findings in aggregated analyses with follow up among more than 6 million young adults from 4 Nordic countries to assess remaining questions and to justify power for analyses of specific NCD-related causes of death. Our aim was to investigate the association between gestational age categories across the entire gestational age spectrum and all-cause and NCD mortality in young adults in Norway, Sweden, Denmark, and Finland.

## Methods

This study was based on individual-level data from nationwide registries in Norway, Sweden, Denmark, and Finland. Data from the medical birth registries (MBRs)^23^ in each country were linked with data from the respective national causes of death registries^24,25,26,27^ using unique identifiers. The study population included all children born alive who were recorded in the Norwegian MBR from 1967 to 2002, the Swedish MBR from 1974 to 2002, the Danish MBR from 1978 to 2001, and the Finnish MBR from 1987 to 1990. For each country, individuals were excluded if their gestational age was outside a plausible range (ie, 23-44 weeks); if their birth weight was greater than 6000 g or less than 350 g; if their birth weight for gestational age was more than 6 SDs from expected^28^; if maternal identity could not be ascertained; or if they died or emigrated before age 15 years. Furthermore, individuals were excluded from the study populations if they had missing information on gestational age, birth weight, sex, birth year, and maternal age. The study followed the Strengthening the Reporting of Observational Studies in Epidemiology (STROBE) reporting guideline.

The Norwegian ethical committee on medical research approved this study; data were delivered and approved by the Norwegian MBR and cause of death registry, both at Norwegian Institute of Public Health.^29^ According to Danish legislation, no ethical permission is required for register-based research; however, this study was approved by the local data protection authorities. This study was approved by the Swedish ethical review board of Stockholm and the research ethics board of the Finnish Institute for Health and Welfare.

Exposure information on gestational age was obtained from the 4 MBRs. In Norway, gestational age was estimated from last menstrual period, ultrasonograph examination, or, in cases of assisted reproduction, from date of embryo transfer. In Sweden, early second-trimester ultrasonography examination has been routinely offered since 1990, and more than 95% of women attended; otherwise, the date of the last menstrual period was used.^30^ In Denmark, gestational age was estimated based on last menstrual period, ultrasonography, or clinical examinations.^31^ In Finland, gestational age was estimated based on last menstrual period and, from the late 1980s onward, confirmed by ultrasonography examination. Gestational age in completed weeks was categorized as moderately preterm or earlier (23-33 weeks), late preterm (34-36 weeks), early term (37-38 weeks), full term (39-41 weeks), and post term (42-44 weeks). However, for underlying causes of death that are rare in this relatively young population (ie, diabetes, lung disease, stroke), all individuals born before 37 weeks were analyzed as 1 preterm group.

Causes and dates of death were obtained from the causes of death registry in each country. Underlying causes of death were registered according to the *International Classification of Disease* (*ICD*), *Eighth*, *Ninth*, and *Tenth* editions, and codes of primary underlying causes of death were grouped according to the European Shortlist for Causes of Death (EU short codes) version 1998.^32^ All-cause mortality and the following primary underlying causes of death were investigated: cardiovascular disease (CVD; EU short codes, 33, 34-36), divided into noncerebrovascular CVD (EU short codes, 33-35) and stroke (EU short code, 36); cancer (EU short codes, 06-24), diabetes (EU short code, 27); chronic lung disease (EU short codes, 40, 41), and NCD (ie, CVD, diabetes, lung disease, and cancer).

Information on birth year, sex, birth weight, congenital malformations, maternal age, and parity was obtained from the national registries. Congenital malformations were defined as having 1 or more congenital malformations registered. Parity was defined as number of previous live births (0 and ≥1).

### Statistical Analysis

Survival time was calculated as time from age 15 years to death, emigration, or end of follow-up (Norway and Sweden, December 31, 2017; Denmark, January 31, 2016; and Finland, December 31, 2014). The Norwegian and Swedish data were pooled and analyzed together; the Danish and Finnish data were analyzed separately and combined in a meta-analysis using the inverse-variance method^33^ for each comparison.^34^ Analyses were performed from June 2019 to May 2020.

For Norwegian and Swedish data, individual data could be pooled and analyzed in Norway. For Denmark and Finland, individual data could not be shared, and analyses had to be performed locally due to restrictions in data sharing regulations. Thus, in Norwegian and Swedish data, more sophisticated analyses, including sibling analyses and cumulative hazards with interaction of sex, could be performed.

Relative risk of mortality in categories of gestational age compared with children born in weeks 39 to 41 was estimated by calculating hazard ratios (HRs) and associated 2-sided 95% Wald type CIs from Cox regression. The HRs were calculated adjusting for potential confounding by maternal age, parity, birth cohort, sex, and birth weight SD scores. Variables were categorized as presented in eTable 1 in the Supplement. Empirical estimates of the cumulative hazard were derived according to Breslow.^35^ In cause-specific modeling, competing risks (ie, death from other causes) were treated as censored.^36^ The proportional hazards assumption was assessed by plotting the log-cumulative hazard against the log of survival time. There was no imputation of missing data.

Sensitivity analyses were performed to assess robustness of main findings. Thus, to explore potential effects from including older populations with longer follow-up in the Norwegian and Swedish data, study populations were restricted to individuals born from 1974 (earliest Swedish cohort) and from 1978 (earliest Danish cohort) in sensitivity analyses. To investigate a potential interaction between sex and gestational age, an interaction term was included in models based on the joint Norwegian and Swedish study populations. To examine familial confounding in the association between gestational age and mortality, sibling analyses were conducted in the Norwegian and Swedish data for the main outcomes (ie, all-cause, NCD, and CVD mortality) using a stratified Cox model, with mother’s identification number defining the strata. Individuals with congenital malformations were excluded in an additional sensitivity analysis. Maternal educational level was included as an additional confounder in sensitivity analyses conducted in the Swedish and Danish study population. All data handling and analyses were performed in SAS version 9.4 (SAS Institute). Statistical significance was set at *P* < .05, and all tests were 2-tailed.

## Results

### Study Population

Among 6 263 941 individuals identified at birth, a total of 6 263 286 individuals (1 910 365 [30.5%] born in Norway, 2 759 206 [44.1%] in Sweden, 1 364 867 [21.8%] in Denmark and 228 848 [3.7%] in Finland) could be followed up after age 15 years. Overall, 339 403 (5.4%) were born preterm, and 3 049 100 (48.7%) were women.

Detailed characteristics of the study population are presented in eTable 1 in the Supplement. Newborn and maternal characteristics were similar across countries, although the Norwegian population included a relatively older population (born in 1985 or earlier: Norway, 1 006 219 [52.7%]; Sweden, 1 094 426 [39.7%]; Denmark, 377 616 [27.7%]). Individuals in the Finnish population had birth years starting in 1987. The moderately preterm and earlier category included 81 727 individuals (1.3%) and the late preterm category 257 676 (4.1%). Distributions of death, emigration, and missing information by gestational age categories are shown in eTable 2 in the Supplement. A larger proportion died before age 15 years in the categories with shorter gestational ages compared with categories closer to full term. Gestational age distribution did not differ substantially between those who emigrated before age 15 years and the study population. Those excluded with missing information was 119 635 (5.7%) in Norway, 16 181 (0.5%) in Sweden, 76 727 (5.3%) in Denmark, and 3797 (1.6%) in Finland, and most of those with missing information lacked information on gestational age.

### Outcomes

During follow-up, 46 936 (0.7%) individuals died. Median (range) age at death was 28.8 (15.0-50.9) years. A total of 10 430 (22.2%) deaths were from NCDs and 2880 (6.2%) deaths were from CVDs. The cause of death was not registered for 608 of 21 706 (2.8%) in Norway, 36 of 18 138 (0.2%) in Sweden, 204 of 5717 (3.6%) in Denmark, and 5 of 1375 (0.4%) in Finland. The most common cause of death category not included in the NCDs studied was external causes of death (accidents and suicides). The median (interquartile range) survival time for those who survived until the end of follow-up was 32.8 (23.6-43.0) years in Norway, 28.7 (22.6-36.3) years in Sweden, 25.5 (20.3-31.5) years in Denmark, and 26.1 (25.1-27.0) years in Finland.

### All-Cause Mortality

**Figure 1.**
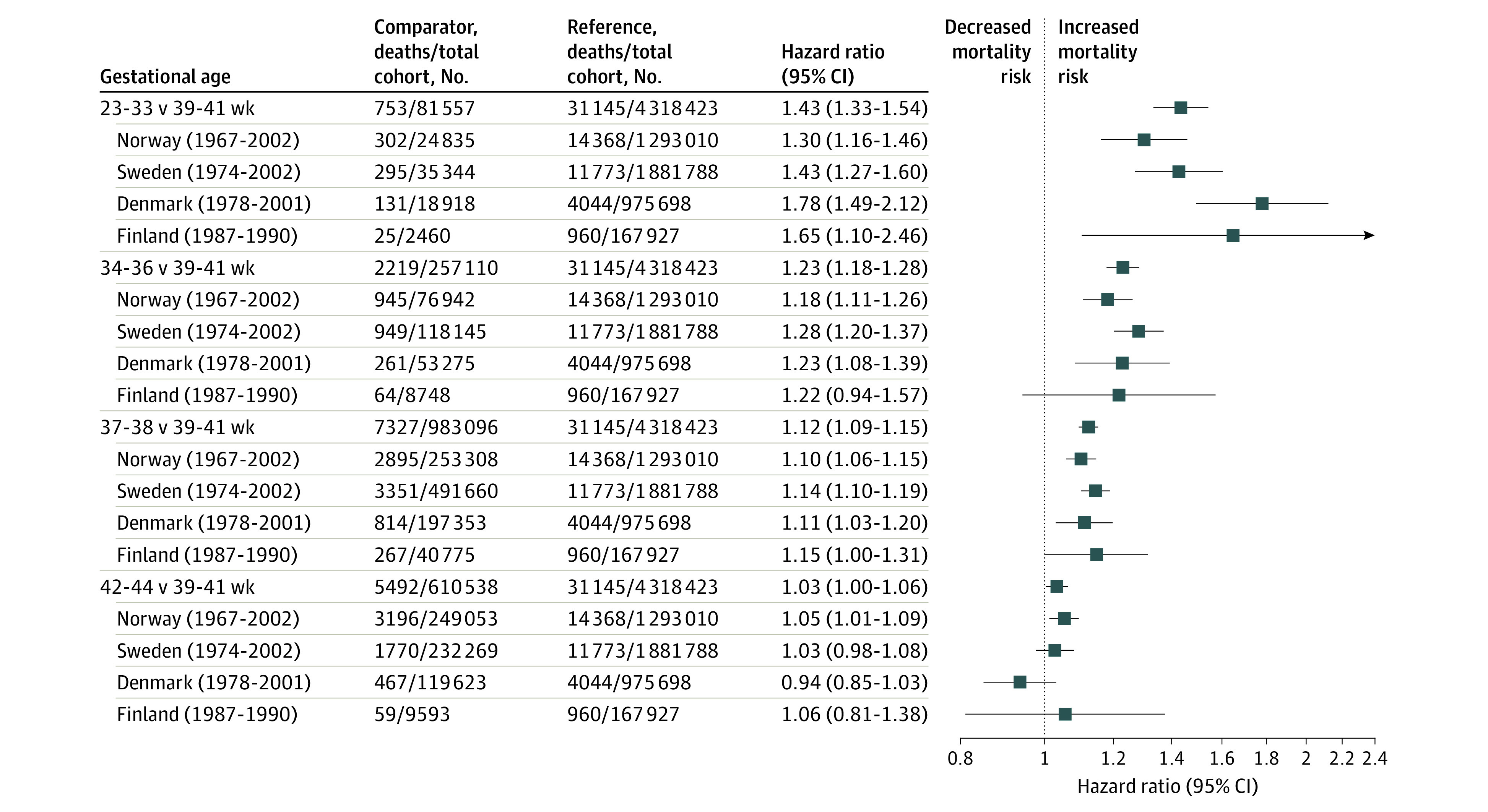
All-Cause Mortality

**Figure 2.**
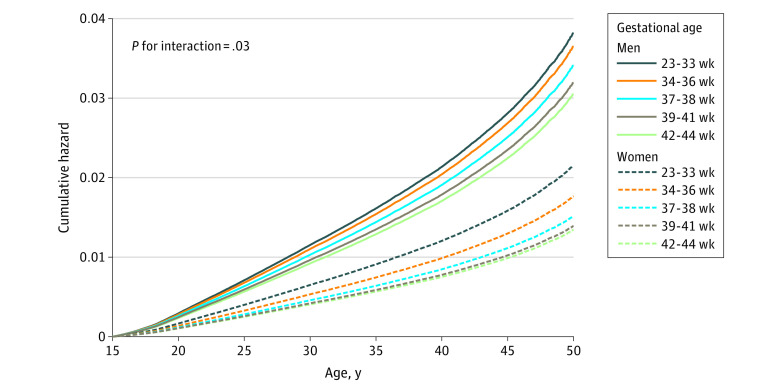
Cumulative Hazard of Death by Sex and Gestational Age Category in Data Sets from Norway and Sweden

The adjusted HR (aHR) for all-cause death after age 15 years in individuals born before week 34 compared with individuals born full term was 1.44 (95% CI, 1.34-1.55); the corresponding aHR for those born moderately preterm and earlier was 1.23 (95% CI, 1.18-1.29), and for those born early term, it was 1.12 (95% CI, 1.09-1.15) (Figure 1). Thus, an inverse dose-response association was found across the preterm and early term gestational age categories. For those born post term, the findings were inconsistent between countries. Cumulative hazard estimates for all-cause mortality in the joint Norwegian and Swedish population are displayed by sex and gestational age categories in Figure 2 and show that total mortality was higher in men compared with women in all gestational age categories. However, the association of gestational age with all-cause mortality were stronger in women than in men (*P* for interaction = .03). The aHRs for all-cause mortality in the moderate preterm group or earlier compared with full term was 1.63 (95% CI, 1.41-1.88) in women and 1.25 (95% CI, 1.13-1.38) in men (eFigure 1 in the Supplement).

### NCD Mortality

**Figure 3.**
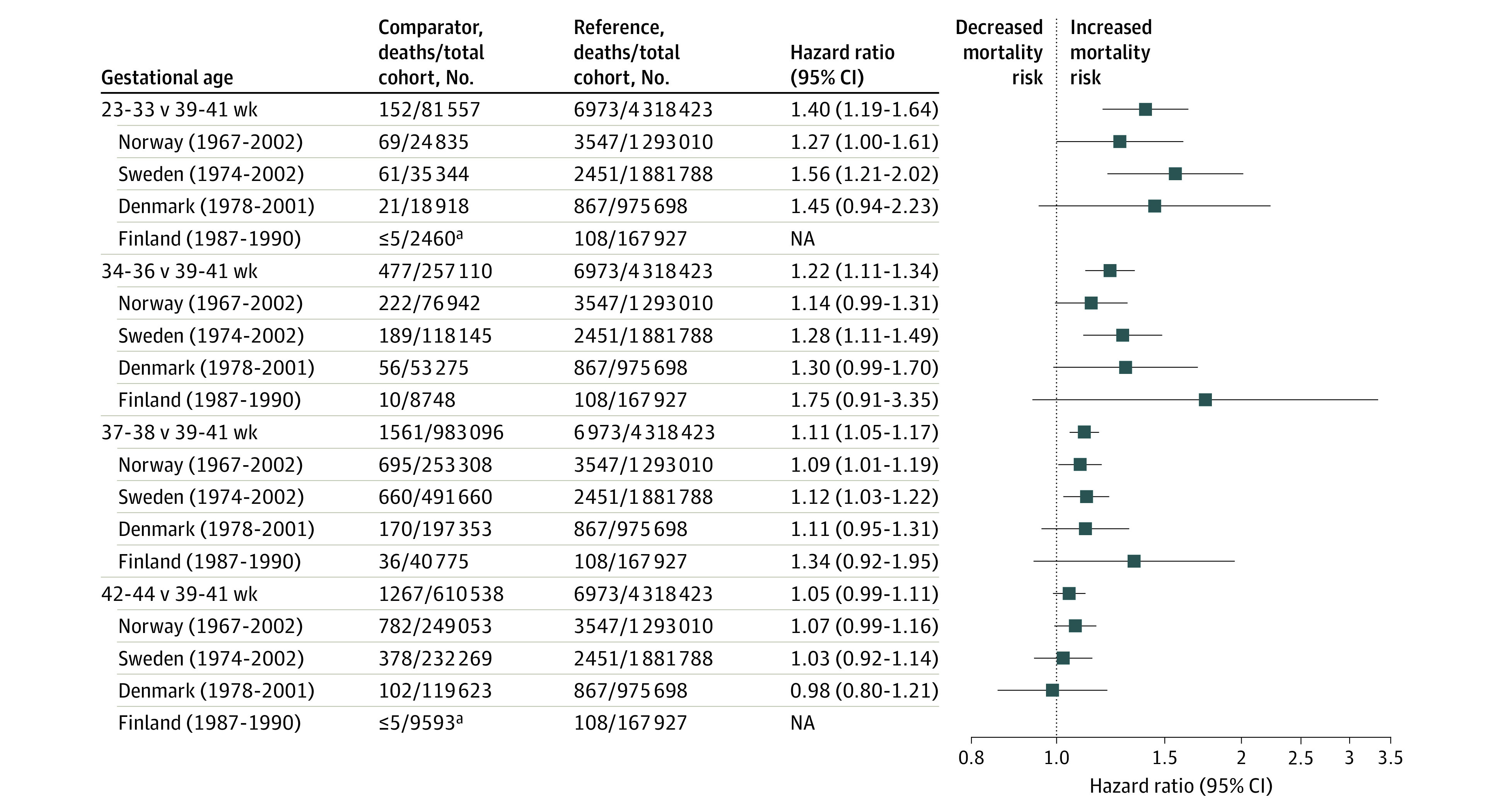
Noncommunicable Disease Mortality NA indicates not applicable.

Results for NCD-related deaths were similar to those for all-cause mortality. A dose-response association across the preterm and early term groups was observed (Figure 3).

Cancer mortality was modestly increased for individuals born moderately preterm and earlier as well as for the postterm group compared with the full-term group. However, precision was low owing to few cancer deaths, especially in the group born moderately preterm and earlier (eFigure 2 in the Supplement).

**Figure 4.**
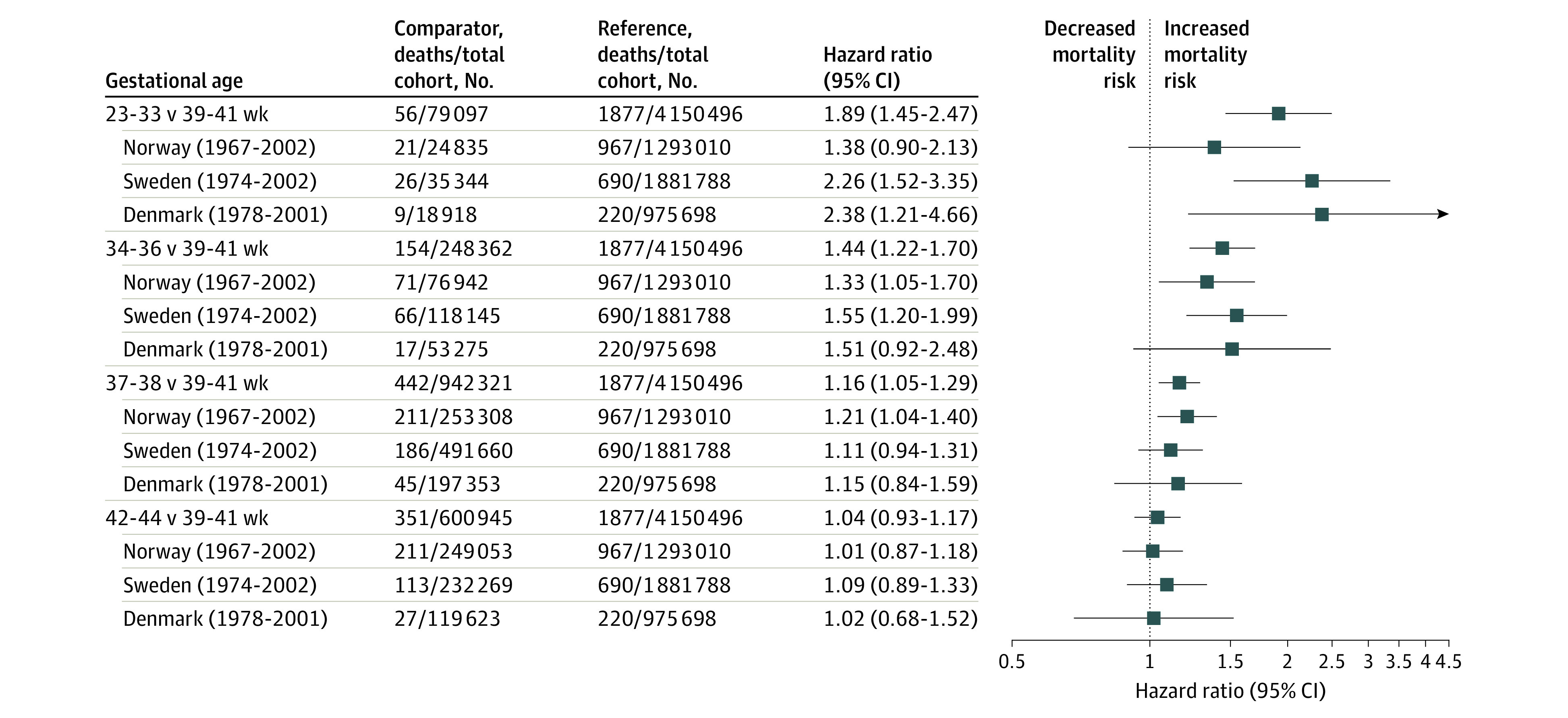
Cardiovascular Disease Mortality

The results for CVD deaths in Norway, Sweden, and Denmark are shown in Figure 4. Finnish numbers were too low to be displayed. Gestational age was inversely associated with CVD mortality, and associations were stronger than those for all-cause and NCD mortality. For those born moderately preterm and earlier, the risk of CVD was nearly twice that of the full-term group (aHR, 1.80; 95% CI, 1.45-2.47).

We assessed associations for mortality from diabetes, chronic lung disease, stroke, and CVDs not related to stroke in the joint Norwegian and Swedish population (Table). The aHRs with 95% CIs for all preterm groups compared with the full-term group were 1.98 (95% CI, 1.44-2.73) for diabetes, 2.28 (95% CI, 1.36-3.82) for chronic lung disease, 1.89 (95% CI, 1.45-2.47) for CVD, and 1.55 (95% CI, 1.31-1.83) for noncerebrovascular CVDs. For stroke, we found no clear association with preterm or early term birth.

**Table.** Associations Between Categories of Gestational Age at Birth and Specific Causes of NCD Deaths, Norwegian and Swedish Data

Cause of death by gestational age, wk^a^	Norway (1967-2002)		Sweden (1974-2002)		Combined	
	Deaths/total No.	aHR (95% CI)^b^	Deaths/total No.	aHR (95% CI)^b^	Deaths/total No.	aHR (95% CI)^b^
**Diabetes**						
23-36	27/101 777	1.96 (1.31-2.93)	16/153 489	2.05 (1.21-3.49)	43/255 266	1.98 (1.44-2.73)
37-38	45/253 308	1.30 (0.94-1.81)	49/491 660	2.04 (1.45-2.88)	94/744 968	1.60 (1.27-2.02)
39-41	189/1 293 010	1 [Reference]	97/1 881 788	1 [Reference]	286/3 174 798	1 [Reference]
42-44	47/249 053	1.13 (0.82-1.56)	13/232 269	0.89 (0.49-1.59)	60/481 322	1.08 (0.81-1.43)
**Chronic lung diseases**						
23-36	11/101 777	2.37 (1.25-4.48)	6/153 489	2.25 (0.94-5.39)	17/255 266	2.28 (1.36-3.82)
37-38	12/253 308	1.00 (0.54-1.85)	10/491 660	1.25 (0.62-2.55)	22/744 968	1.10 (0.69-1.75)
39-41	68/1 293 010	1 [Reference]	33/1 881 788	1 [Reference]	101/3 174 798	1 [Reference]
42-44	11/249 053	0.72 (0.38-1.37)	4/232 269	0.78 (0.27-2.21)	15/481 322	0.74 (0.43-1.27)
**Stroke**						
23-36	18/101 777	1.31 (0.81-2.13)	9/153 489	1.02 (0.52-2.02)	27/255 266	1.21 (0.81-1.79)
37-38	27/253 308	0.78 (0.52-1.16)	21/491 660	0.82 (0.51-1.31)	48/744 968	0.80 (0.59-1.08)
39-41	199/1 293 010	1 [Reference]	107/1 881 788	1 [Reference]	306/3 174 798	1 [Reference]
42-44	42/249 053	0.95 (0.68-1.34)	18/232 269	1.03 (0.62-1.70)	60/481 322	0.97 (0.73-1.28)
**Other CVD**						
23-36	74/101 777	1.34 (1.05-1.70)	83/153 489	1.82 (1.45-2.30)	157/255 266	1.55 (1.31-1.83)
37-38	184/253 308	1.31 (1.12-1.54)	165/491 660	1.16 (0.97-1.38)	349/744 968	1.24 (1.10-1.39)
39-41	768/1 293 010	1 [Reference]	583/1 881 788	1 [Reference]	1351/3 174 798	1 [Reference]
42-44	169/249 053	1.05 (0.89-1.25)	95/232 269	1.08 (0.87-1.35)	264/481 322	1.06 (0.93-1.21)

Abbreviations: aHR, adjusted hazard ratio; CVD, cardiovascular disease.

^a^ Causes of death determined by European Shortlist for Causes of Death, as follows: diabetes, code 27; chronic lung disease, codes 40 and 41; stroke, code 36; other CVD, codes 33 to 35. Cause-specific hazard^36^ was calculated.

^b^ Cox regression adjusting for birth cohort (≤1985, >1985), sex, birth weight (SD), and maternal parity (0, ≥1).

### Sensitivity Analyses

There were no indications of nonproportional hazards. Sensitivity analyses excluding congenital malformations (eTable 3 in the Supplement) confirmed that higher mortality in the preterm group remained after exclusion. In the Swedish (born after 1990) and Danish (born after 1978) data, adjustment for maternal education marginally attenuated the estimates. In the Danish data, the aHR for all-cause mortality in those born before 34 weeks vs those born full term was 1.71 (95% CI, 1.43-2.05) when maternal education was included (data not shown).

We explored the potential effect of the Norwegian and Swedish cohorts being older than the other cohorts by including only the later birth years in the follow-up (ie, all born after 1974, which was the start of the Swedish MBR, and all born after 1978, which was the start of the Danish MBR) (eFigures 3-9 in the Supplement). Results show similar associations as the complete follow-up. For CVD mortality, both the overall estimate and the Norwegian estimate for those born moderately preterm and earlier increased (aHR, 2.22; 95% CI, 1.65-2.98; aHR, 2.00; 95% CI, 1.09-2.65, respectively, when excluding the earliest birth years).

Additional analyses in the Norwegian and Swedish data for comparisons within sibling groups, using maternal identity as strata, showed similar patterns across the categories as in the main analyses, but with some attenuation of estimated HRs and with lower precision (eTable 4 in the Supplement). Thus, in the sibling analysis, the aHR for all causes of deaths for individuals born moderately preterm or earlier was 1.34 (95% CI, 1.14-1.57), the corresponding aHRs were 1.29 (95% CI, 0.90-1.84) for NCD and 1.75 (95% CI, 0.91-3.34) for CVD.

## Discussion

Adults born preterm or early term were at increased risk of death from all causes and NCDs, and findings were replicated across 4 Nordic countries. Importantly, the association was not restricted to the group with the lowest gestational age but was observed across all groups born before full-term gestation, including the much larger early-term group, who were born close to the ideal timing of birth. The association of gestational age with all-cause mortality was stronger in women. The associations could not be explained by individual confounding factors, genetic and environmental factors shared between family members, congenital malformations, or socioeconomic factors. Mortality from NCD and from specific categories of chronic diseases showed similar patterns as for all-cause mortality, with a 2-fold higher risk of mortality from CVD, diabetes, and chronic lung disease for individuals born preterm and with increased risk of mortality from CVD or diabetes in individuals born early term compared with those born full term.

Swedish studies from 2011^18^ and 2013^20^ showed an inverse association between increasing gestational age and mortality in young adulthood. A reanalysis^19^ showed that preterm birth was associated with 40% increased all-cause mortality in the group aged 20 to 29 years and 30% increase in the group aged 30 to 42 years. In a Norwegian study^21^ with follow-up to 2011 (ie, maximum of 45 years of age) similar results were observed. In the current study, we pooled updated Norwegian and Swedish data through 2017 and included Danish and Finnish birth cohorts in a meta-analysis approach to present the largest study to date to our knowledge, with a maximal follow-up until 50 years of age, to assess remaining questions and to justify power for analyses of specific NCD-related causes of death. The results are mainly homogenous across the countries displayed. Our data support the finding that the risk of premature death in individuals born preterm compared with those born full term is higher in women than in men. Because women with cardiometabolic risk patterns and less favorable socioeconomic conditions are at a higher risk of both pregnancy complications and preterm birth,^37^ it could seem plausible that the association of shorter gestation with adult mortality could be confounded by maternal and socioeconomic factors. Similarly, offspring with congenital malformations are more frequently born before term and have long-term morbidities that may influence mortality.^38,39^ Nevertheless, both the current and previous studies that have been able to take these factors into account^18,19,21^ indicate that the increased adult mortality in individuals born after shorter gestation cannot be attributed to any of these factors. A tendency of marginally strengthened estimates associated with preterm birth if birth years are restricted to births after the late 1970s (rather than including the late 1960s and the early 1970s) was evident for CVD. Weaker estimates for adult mortality in the groups with the most preterm birth in the earliest birth cohorts could be explained by lower follow-up rates due to higher child mortality in this group. Also, a higher proportion of gestational age estimations based on last menstrual period (rather than based on ultrasonography) in the earlier cohorts may decrease precision and thus attenuate associations for the earliest years.

### Strengths and Limitations

In the current analyses, we have overcome regulatory barriers that often preclude aggregated analyses of sensitive registry data across countries.^40^ By pooling analyses across the Nordic countries, we are in a unique position to address limitations of previous studies on surviving individuals who were born preterm, are still relatively young, and for whom death from NCDs is still a relatively rare outcome. The longitudinal nature of the Nordic registries and the opportunity to link data between the national registries enabled us to follow up individuals from birth into adulthood (to a maximum of 50 years) in 4 Nordic countries. The 4 large populations allowed analyses to include specific groups of NCD mortality. The use of nationwide population registries also reduced selection and ascertainment biases. The data from Norway and Sweden were combined on an individual level, and this enabled the investigation of familial confounding, the investigation of interactions between sex and degree of prematurity, and the investigation of rare causes of death. Findings from the Swedish and Danish population, where information on maternal education was available, did not suggest that socioeconomic status could explain the association of gestational age with adult mortality.

There are important limitations to the study as well. The cohort is still relatively young, and despite a large sample size, the number of outcomes limited assessment of more narrowly defined preterm categories for the specific outcomes. Furthermore, different methods of estimating gestational age may have influenced the accuracy of the estimates of gestational age. Compared with estimates of gestational age based on last menstrual period, it has been found that estimates based on ultrasonography shifted the distribution of gestational age lower.^41^ Consequently, this could have produced less contrast and more conservative estimates, particularly in the earlier years of the study period, when ultrasonography was not used routinely. Lower neonatal and childhood survival in the earliest birth cohort may have introduced survivor bias, with only the healthiest reaching adulthood. Neonatal survival and treatment has changed substantially from the beginning of the study period^42^; therefore, it is unclear to what extent our findings are generalizable to children born today and to children in other settings with fewer resources. It remains to be observed whether these patterns of higher mortality in individuals born before term persist later in adulthood and whether they apply to more recent birth cohorts with increased neonatal survival.

## Conclusions

The current findings add evidence of higher risk of young adult death from all causes and from chronic diseases in individuals born before full gestation. Importantly, the altered risk included the late preterm and early-term groups, who were born close to ideal birth timing. This may have implications not only for tailoring chronic disease risk assessments in individuals born before full term but also for the interpretation of the rich literature regarding the association between birth weight and mortality, in which gestational age has not been taken into account.

## Notes

Supplement. **eTable 1. **Baseline Characteristics by Gestational Age Categories in 4 Nordic Countries **eTable 2. **Excluded and Included Individuals According to Gestational Age by Country **eFigure 1. **Hazard Ratio and 95% CI for Sex Differences in All-Cause Mortality and Noncommunicable Disease Mortality According to Gestat ional Age Categories for Norway and Sweden Combined **eFigure 2. **Cancer Mortality by Country and Overall: Hazard Ratios (HRs) and 95% CIs by Categories of Gestational Age **eTable 3. **Mortality From Specific NCDs by Country and Overall, Excluding Individuals With Congenital Anomalies, Hazard Ratios (HRs) and 95% CIs by Categories of Gestational Age **eFigure 3. **All-Cause Mortality by Country and Overall, Restricting Birth Year to 1974 and Onward, Hazard Ratios (HRs) and 95% CIs by Categories of Gestational Age **eFigure 4. **All-Cause Mortality by Country and Overall, Restricting Birth Year to 1978 and Onwards, Hazard Ratios (HRs) and 95% CIs by Categories of Gestational Age **eFigure 5. **All-Cause Mortality by Country and Overall, Restricting Birth Year to 1978 and Onwards, Hazard Ratios (HRs) and 95% CIs by Categories of Gestational Age **eFigure 6. **Noncommunicable Disease Mortality by Country and Overall, Restricting Birth Year to 1974 and Onward, Hazard Ratios (HRs) and 95% CIs by Categories of Gestational Age **eFigure 7. **Noncommunicable Disease Mortality by Country and Overall, Restricting Birth Year to 1978 and Onwards, Hazard Ratios (HRs) and 95% CIs by Categories of Gestational Age **eFigure 8. **Cardiovascular Disease Mortality by Country and Overall, Restricting Birth Year to 1974 and Onward, Hazard Ratios (HRs) and 95% CIs by Categories of Gestational Age **eFigure 9. **Cardiovascular Disease Mortality by Country and Overall, Restricting Birth Year to 1978 and Onwards, Hazard Ratios (HRs) and 95% CIs by Categories of Gestational Age **eTable 4. **Hazard Ratio (95% CI) for Sibling Analysis
