# Errors in Key Points, Results, and Supplement

**DOI:** 10.1001/jamanetworkopen.2021.0068

**Published:** 2021-02-05

In the Original Investigation titled “Mortality Among Young Adults Born Preterm and Early Term in 4 Nordic Nations,”^1^ published January 8, 2021, there were errors in the Key Points, Results, and Supplement. The study included more than 6.2 million individuals. The first sentence of the Results section should have stated that 6 665 429 individuals were identified at birth. In the Supplement, eTable 2 has been replaced with a corrected version. This article has been corrected.^1^
