# Supplementary material for: Mortality Among Young Adults Born Preterm and Early Term in 4 Nordic Nations
Source: JAMA Netw Open. 2021 Jan 8;4(1):e2032779. doi: 10.1001/jamanetworkopen.2020.32779 (PMC7794670; doi:10.1001/jamanetworkopen.2020.32779)
Supplement: Supplement. — eTable 1. Baseline Characteristics by Gestational Age Categories in 4 Nordic Countries eTable 2. Excluded and Included Individuals According to Gestational Age by Country eFigure 1. Hazard Ratio and 95% CI for Sex Differences in All-Cause Mortality and Noncommunicable Disease Mortality According to Gestat ional Age Categories for Norway and Sweden Combined eFigure 2. Cancer Mortality by Country and Overall: Hazard Ratios (HRs) and 95% CIs by Categories of Gestational Age eTable 3. Mortality From Specific NCDs by Country and Overall, Excluding Individuals With Congenital Anomalies, Hazard Ratios (HRs) and 95% CIs by Categories of Gestational Age eFigure 3. All-Cause Mortality by Country and Overall, Restricting Birth Year to 1974 and Onward, Hazard Ratios (HRs) and 95% CIs by Categories of Gestational Age eFigure 4. All-Cause Mortality by Country and Overall, Restricting Birth Year to 1978 and Onwards, Hazard Ratios (HRs) and 95% CIs by Categories of Gestational Age eFigure 5. All-Cause Mortality by Country and Overall, Restricting Birth Year to 1978 and Onwards, Hazard Ratios (HRs) and 95% CIs by Categories of Gestational Age eFigure 6. Noncommunicable Disease Mortality by Country and Overall, Restricting Birth Year to 1974 and Onward, Hazard Ratios (HRs) and 95% CIs by Categories of Gestational Age eFigure 7. Noncommunicable Disease Mortality by Country and Overall, Restricting Birth Year to 1978 and Onwards, Hazard Ratios (HRs) and 95% CIs by Categories of Gestational Age eFigure 8. Cardiovascular Disease Mortality by Country and Overall, Restricting Birth Year to 1974 and Onward, Hazard Ratios (HRs) and 95% CIs by Categories of Gestational Age eFigure 9. Cardiovascular Disease Mortality by Country and Overall, Restricting Birth Year to 1978 and Onwards, Hazard Ratios (HRs) and 95% CIs by Categories of Gestational Age eTable 4. Hazard Ratio (95% CI) for Sibling Analysis [file jamanetwopen-e2032779-s001.pdf]

Supplemental Online Content

Risnes K, Bilsteen JF, Brown P, et al. Mortality among young adults born preterm and early term in 4 Nordic nations. *JAMA Netw Open*. 2021;4(1):e2032779. doi:10.1001/jamanetworkopen.2020.32779

eTable 1. Baseline Characteristics by Gestational Age Categories in 4 Nordic Countries
eTable 2. Excluded and Included Individuals According to Gestational Age by Country
eFigure 1. Hazard Ratio and 95% CI for Sex Differences in All-Cause Mortality and Noncommunicable Disease Mortality According to Gestat ional Age Categories for Norway and Sweden Combined
eFigure 2. Cancer Mortality by Country and Overall: Hazard Ratios (HRs) and 95% CIs by Categories of Gestational Age
eTable 3. Mortality From Specific NCDs by Country and Overall, Excluding Individuals With Congenital Anomalies, Hazard Ratios (HRs) and 95% CIs by Categories of Gestational Age
eFigure 3. All-Cause Mortality by Country and Overall, Restricting Birth Year to 1974 and Onward, Hazard Ratios (HRs) and 95% CIs by Categories of Gestational Age
eFigure 4. All-Cause Mortality by Country and Overall, Restricting Birth Year to 1978 and Onwards, Hazard Ratios (HRs) and 95% CIs by Categories of Gestational Age
eFigure 5. All-Cause Mortality by Country and Overall, Restricting Birth Year to 1978 and Onwards, Hazard Ratios (HRs) and 95% CIs by Categories of Gestational Age
eFigure 6. Noncommunicable Disease Mortality by Country and Overall, Restricting Birth Year to 1974 and Onward, Hazard Ratios (HRs) and 95% CIs by Categories of Gestational Age
eFigure 7. Noncommunicable Disease Mortality by Country and Overall, Restricting Birth Year to 1978 and Onwards, Hazard Ratios (HRs) and 95% CIs by Categories of Gestational Age
eFigure 8. Cardiovascular Disease Mortality by Country and Overall, Restricting Birth Year to 1974 and Onward, Hazard Ratios (HRs) and 95% CIs by Categories of Gestational Age
eFigure 9. Cardiovascular Disease Mortality by Country and Overall, Restricting Birth Year to 1978 and Onwards, Hazard Ratios (HRs) and 95% CIs by Categories of Gestational Age
eTable 4. Hazard Ratio (95% CI) for Sibling Analysis

This supplemental material has been provided by the authors to give readers additional information about their work.

© 2021 Risnes K et al. *JAMA Network Open.*

*eTable 1. Baseline Characteristics by Gestational Age Categories in 4 Nordic Countries**

		Categories of gestation (weeks)					
Characteristic		23-33	34-36	37-38	39-41	42-44	Overall
Norway							
Birth year	<=1985	10,331 (41.3)	37,035 (47.8)	121,612 (47.6)	694,475 (53.3)	142,766 (57.0)	1,006,219 (52.7)
	>1985	14,680 (58.7)	40,501 (52.2)	133,661 (52.4)	607,508 (46.7)	107,796 (43.0)	904,146 (47.3)
Sex	Male	13,625 (54.5)	42,509 (54.8)	138,425 (54.2)	660,257 (50.7)	125,285 (50.0)	980,101 (51.3)
Birth weight	(gram)	1780 (1390,2160)	2728 (2380,3120)	3240 (2930,3560)	3600 (3290,3915)	3730 (3400,4060)	3540 (3200,3880)
	(SD)	-0.78 (-1.79, 0.35)	-0.13 (-1.14, 1.05)	0.00 (-0.83, 0.87)	-0.14 (-0.88, 0.62)	-0.65 (-1.39, 0.12)	-0.19 (-0.97, 0.60)
Maternal age	<=24	8,154 (32.6)	26,661 (34.4)	79,884 (31.3)	435,922 (33.5)	100,384 (40.1)	651,005 (34.1)
	25-29	8,019 (32.1)	24,798 (32.0)	85,024 (33.3)	463,238 (35.6)	86,739 (34.6)	667,818 (35.0)
	30-35	6,551 (26.2)	19,256 (24.8)	67,270 (26.4)	316,236 (24.3)	51,017 (20.4)	460,330 (24.1)
	>=36	2,287 (9.1)	6,821 (8.8)	23,095 (9.0)	86,587 (6.7)	12,422 (5.0)	131,212 (6.9)
Previous births	0	12,512 (50.4)	36,367 (47.3)	101,398 (40.0)	517,436 (40.0)	116,876 (46.9)	784,589 (41.4)
	>=1	12,323 (49.6)	40,575 (52.7)	151,910 (60.0)	775,574 (60.0)	132,177 (53.1)	1,112,559 (58.6)
Sweden							
Birth year	<=1985	11,107 (31.4)	44,565 (37.7)	179,468 (36.5)	744,334 (39.6)	114,952 (49.5)	1,094,426 (39.7)
	>1985	24,237 (68.6)	73,580 (62.3)	312,192 (63.5)	1,137,454 (60.4)	117,317 (50.5)	1,664,780 (60.3)
Sex	Male	19,254 (54.5)	63,803 (54.0)	254,393 (51.7)	955,819 (50.8)	123,770 (53.3)	1,417,039 (51.4)
Birth weight	(gram)	1730 (1344,2070)	2685 (2375,2991)	3240 (2950,3545)	3610 (3310,3920)	3820 (3500,4145)	3530 (3181,3870)
	(SD)	-0.86 (-1.83, 0.05)	-0.26 (-1.15, 0.62)	0.01 (-0.76, 0.82)	-0.07 (-0.79, 0.67)	-0.40 (-1.12, 0.33)	-0.10 (-0.84, 0.67)
Maternal age	<=24	8,994 (25.4)	31,383 (26.6)	118,863 (24.2)	482,818 (25.7)	66,062 (28.4)	708,120 (25.7)
	25-29	11,458 (32.4)	40,114 (34.0)	172,218 (35.0)	704,332 (37.4)	86,034 (37.0)	1,014,156 (36.8)
	30-35	10,726 (30.3)	34,174 (28.9)	148,036 (30.1)	546,001 (29.0)	63,632 (27.4)	802,569 (29.1)
	>=36	4,166 (11.8)	12,474 (10.6)	52,543 (10.7)	148,637 (7.9)	16,541 (7.1)	234,361 (8.5)
Previous births	0	16,249 (46.0)	53,328 (45.1)	190,443 (38.7)	765,788 (40.7)	111,921 (48.2)	1,137,729 (41.2)
	>=1	19,095 (54.0)	64,817 (54.9)	301,217 (61.3)	1,116,000 (59.3)	120,348 (51.8)	1,621,477 (58.8)
Denmark							
Birth year	<=1985	4,303 (22.7)	13,216 (24.8)	46,098 (23.4)	281,679 (28.9)	32,320 (27.0)	377,616 (27.7)
	>1985	14,615 (77.3)	40,059 (75.2)	151,255 (76.6)	694,019 (71.1)	87,303 (73.0)	987,251 (72.3)
Sex	Male	10,363 (54.8)	29,005 (54.4)	104,416 (52.9)	494,220 (50.7)	61,930 (51.8)	699,934 (51.3)
Birth weight	(gram)	1640 (1300, 1980)	2535 (2220, 2858)	3120 (2810, 3450)	3520 (3216, 3850)	3750 (3440, 4090)	3470 (3100, 3800)
	(SD)	-0.9 (-1.9, 0.0)	-0.5 (-1.4, 0.5)	-0.1 (-0.9, 0.8)	-0.1 (-0.9, 0.7)	-0.5 (-1.2,0.3)	-0.2 (-1.0, 0.6)
Maternal age	<=24	4,543 (24.0)	13,562 (25.5)	47,072 (23.9)	238,709 (24.5)	30,440 (25.4)	334,326 (24.5)
	25-29	6,670 (35.3)	19,577 (36.7)	72,667 (36.8)	387,041 (39.7)	48,108 (40.2)	534,063 (39.1)
	30-35	5,837 (30.9)	15,357 (28.8)	60,069 (30.4)	284,619 (29.2)	33,979 (28.4)	399,861 (29.3)
	>=36	1,868 (9.9)	4,779 (9.0)	17,545 (8.9)	65,329 (6.7)	7,096 (5.9)	96,617 (7.1)
Previous births	0	10,380 (54.9)	27,766 (52.1)	87,045 (44.1)	431,503 (44.2)	60,920 (50.9)	617,614 (45.3)
	>=1	8,538 (45.1)	25,509 (47.9)	110,308 (55.9)	544,195 (55.8)	58,703 (49.1)	747,253 (54.7)
Finland							
Birth year	>1985	2,460 (100.0)	8,748 (100.0)	40,775 (100.0)	167,927 (100.0)	9,593 (100.0)	229,503 (100.0)
Sex	Male	1,375 (55.9)	4,705 (53.8)	21,485 (52.7)	84,925 (50.6)	4,965 (51.8)	117,455 (51.2)
Birth weight	(gram)	1680 (1310, 2060)	2720 (2390, 3050)	3310 (3010, 3620)	3665 (3370, 3970)	3840 (3550, 4150)	3580 (3250, 3915)
	(SD)	-0.80 (-1.81, 0.11)	-0.17 (-1.11, 0.80)	0.20 (-0.59, 1.04)	0.09 (-0.62, 0.83)	-0.26 (-0.96, 0.42)	0.08 (-0.66, 0.84)
Maternal age	<=24	545 (22.2)	2,107 (24.1)	8,715 (21.4)	38,323 (22.8)	2,480 (25.9)	52,170 (22.7)
	25-29	799 (32.5)	2,868 (32.8)	14,428 (35.4)	63,981 (38.1)	3,739 (39.0)	85,815 (37.4)

(Continued)

© 2021 Risnes K et al. *JAMA Network Open.*

eTable 1. Baseline Characteristics

		Categories of gestation (weeks)					
Characteristic		23-33	34-36	37-38	39-41	42-44	Overall
	30-35	744 (30.2)	2,613 (29.9)	12,517 (30.7)	49,827 (29.7)	2,719 (28.3)	68,420 (29.8)
	>=36	372 (15.1)	1,160 (13.3)	5,115 (12.5)	15,796 (9.4)	655 (6.8)	23,098 (10.1)
Previous births	0	1,224 (49.9)	3,978 (45.6)	15,488 (38.1)	65,658 (39.2)	5,110 (53.5)	91,458 (40.0)
	>=1	1,230 (50.1)	4,742 (54.4)	25,176 (61.9)	101,803 (60.8)	4,439 (46.5)	137,390 (60.0)
SD: standard deviation. For continuous variables (age, weight) the median (Q1,Q3) is displayed.							

*The study population included all liveborn children recorded in the Norwegian MBR from 1967 to 2002, the Swedish MBR from 1974 to 2002, the Danish MBR from 1978 to 2001 and the Finnish MBR from 1987 to 1990.

© 2021 Risnes K et al. *JAMA Network Open.*

*eTable 2. Excluded and Included Individuals According to Gestational Age by Country**

	Gestational age (weeks)							
	23-33	34-36	37-38	39-41	42-45	<23 or >45	Missing GA	overall
**Norway n (%)**								
Study population	34036 (1.7)	80187 (4.1)	259313 (13.2)	1315670 (67.0)	253293 (12.9)	19829 (1.0)	0 (0.0)	1,962,328
Dead before 15 years	5963 (24.8)	1963 (8.2)	2764 (11.5)	9112 (37.9)	2046 (8.5)	593 (2.5)	1588 (6.6)	24,029
Emigrated before 15 years	91 (1.8)	217 (4.3)	864 (17.0)	3261 (64.3)	437 (8.6)	38 (0.7)	164 (3.2)	5072
Missing information	2952 (2.5)	458 (0.4)	385 (0.3)	1202 (1.0)	229 (0.2)	916 (0.8)	113493 (94.9)	119,635
**Sweden n (%)**								
Study population	43986 (1.5)	125743 (4.4)	514476 (17.8)	1962341 (67.9)	242769 (8.4)	132 (0.0)	0 (0.0)	2889447
Dead before 15 years	6177 (27.3)	2548 (11.3)	3610 (15.9)	8612 (38.0)	1337 (5.9)	116 (0.5)	242 (1.1)	22642
Emigrated before 15 years	1223 (1.2)	4177 (4.2)	17605 (17.7)	67451 (67.9)	8603 (8.7)	0 (0.0)	336 (0.3)	99395
Missing information	1238 (7.7))	871 (5.4)	1597 (9.9)	4482 (27.7)	561 (3.5)	9 (0.1)	7423 (45.9)	16181
**Denmark n (%)**								
Study population	18918 (1.4)	53275 (3.9)	197353 (14.5)	975698 (71.5)	119623 (8.8)	69 (0.0)	0 (0.0)	1364936
Dead before 15 years	3473 (25.2)	1242 (9.0%)	1837 (13.3)	5569 (40.4)	719 (5.2)	117 (0.9)	842 (6.1)	13799
Emigrated before 15 years	251 (1.2)	761 (3.4)	3144 (15.2)	13896 (67.3)	1653 (8.0)	0 (0.0)	954 (4.6)	20659
Missing information	619 (0.8)	744 (1.0)	1832 (2.4)	6074 (7.9)	867 (1.1)	39 (0.1)	66483 (86.3)	76658
**Finland n (%)**								
Study population	2444 (1.1)	8668 (3.8)	40380 (17.8)	166309 (73.2)	9480 (4.2)	0 (0.0)	0 (0.0)	228848
Dead before 15 years	551 (27.9)	191 (9.7)	311 (15.8)	797 (40.4)	44 (2.2)	27 (1.4)	52 (2.6)	1973
Emigrated before 15 years	9 (0.9)	37 (3.7)	165 (16.4)	711 (70.7)	49 (4.9)	0 (0.0)	35 (3.5)	1006
Missing information	109 (2.9)	58 (1.5)	135 (3.6)	496 (13.1)	46 (1.2)	0 (0.0)	2953 (77.8)	3797

Study population did not include individuals with a gestational age <23 or >45 weeks of gestation.
Missing information included individuals with missing or implausible information on gestational age, birth weight or parity.
*The study population included all liveborn children recorded in the Norwegian MBR from 1967 to 2002, the Swedish MBR from 1974 to 2002, the Danish MBR from 1978 to 2001 and the Finnish MBR from 1987 to 1990.

© 2021 Risnes K et al. *JAMA Network Open.*

eFigure 1. Hazard Ratio and 95% CI for Sex Differences in All-Cause Mortality and Noncommunicable Disease Mortality According to Gestational Age Categories for Norway and Sweden Combined

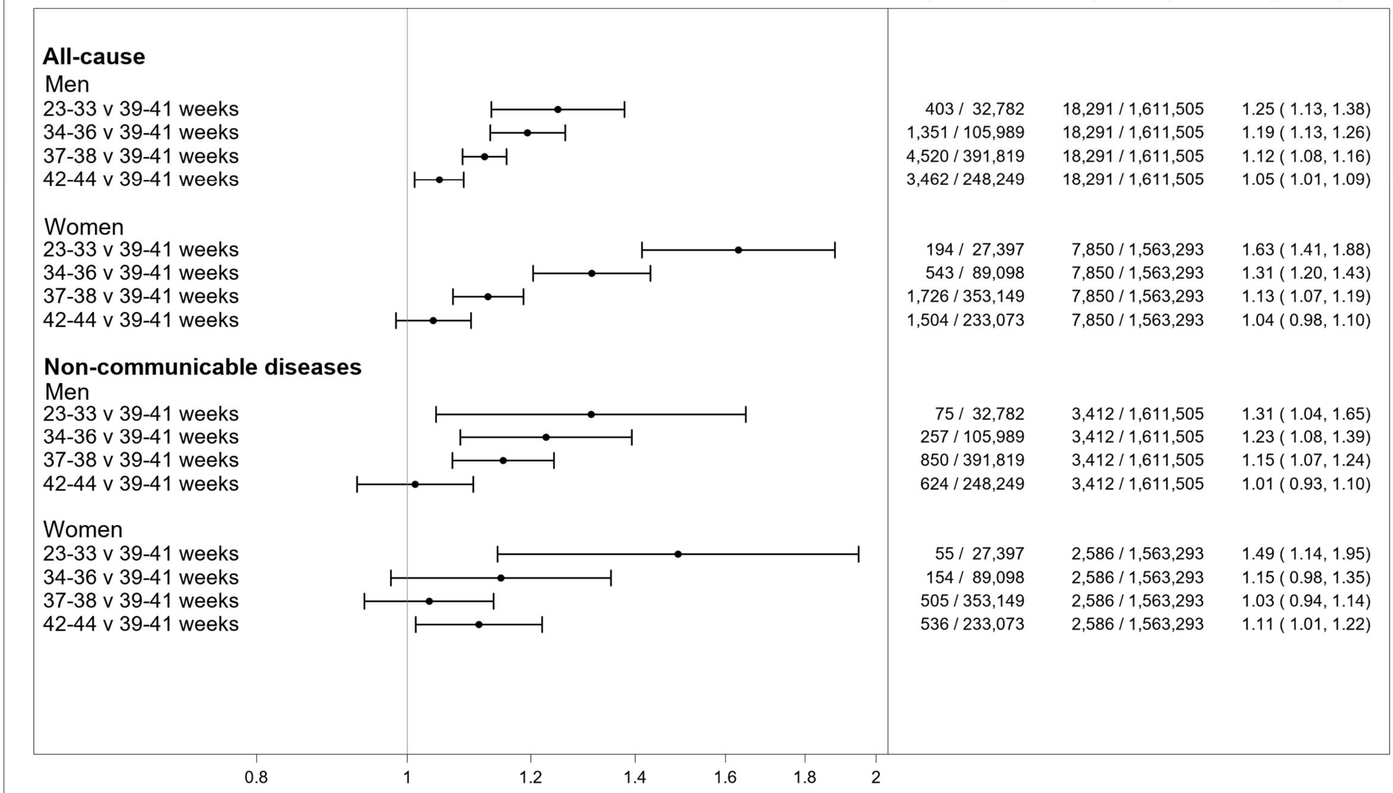

	Comparator (deaths/n)	Reference (deaths/n)	Hazard Ratio (95% CI)
**All-cause**			
Men			
23-33 v 39-41 weeks	403 / 32,782	18,291 / 1,611,505	1.25 (1.13, 1.38)
34-36 v 39-41 weeks	1,351 / 105,989	18,291 / 1,611,505	1.19 (1.13, 1.26)
37-38 v 39-41 weeks	4,520 / 391,819	18,291 / 1,611,505	1.12 (1.08, 1.16)
42-44 v 39-41 weeks	3,462 / 248,249	18,291 / 1,611,505	1.05 (1.01, 1.09)
Women			
23-33 v 39-41 weeks	194 / 27,397	7,850 / 1,563,293	1.63 (1.41, 1.88)
34-36 v 39-41 weeks	543 / 89,098	7,850 / 1,563,293	1.31 (1.20, 1.43)
37-38 v 39-41 weeks	1,726 / 353,149	7,850 / 1,563,293	1.13 (1.07, 1.19)
42-44 v 39-41 weeks	1,504 / 233,073	7,850 / 1,563,293	1.04 (0.98, 1.10)
**Non-communicable diseases**			
Men			
23-33 v 39-41 weeks	75 / 32,782	3,412 / 1,611,505	1.31 (1.04, 1.65)
34-36 v 39-41 weeks	257 / 105,989	3,412 / 1,611,505	1.23 (1.08, 1.39)
37-38 v 39-41 weeks	850 / 391,819	3,412 / 1,611,505	1.15 (1.07, 1.24)
42-44 v 39-41 weeks	624 / 248,249	3,412 / 1,611,505	1.01 (0.93, 1.10)
Women			
23-33 v 39-41 weeks	55 / 27,397	2,586 / 1,563,293	1.49 (1.14, 1.95)
34-36 v 39-41 weeks	154 / 89,098	2,586 / 1,563,293	1.15 (0.98, 1.35)
37-38 v 39-41 weeks	505 / 353,149	2,586 / 1,563,293	1.03 (0.94, 1.14)
42-44 v 39-41 weeks	536 / 233,073	2,586 / 1,563,293	1.11 (1.01, 1.22)

CI: confidence interval. n displayed is the number of individuals included in the analysis, i.e. those with no missing covariate information
Cox regression adjusting for country, birth cohort (<=1985, >1985), sex, birth weight (SD), maternal parity (0,>=1). Cause-specific hazard (Wolders et al. EHJ 2014)

© 2021 Risnes K et al. *JAMA Network Open.*

eFigure 2. Cancer Mortality by Country and Overall: Hazard Ratios (HRs) and 95% CIs by Categories of Gestational Age

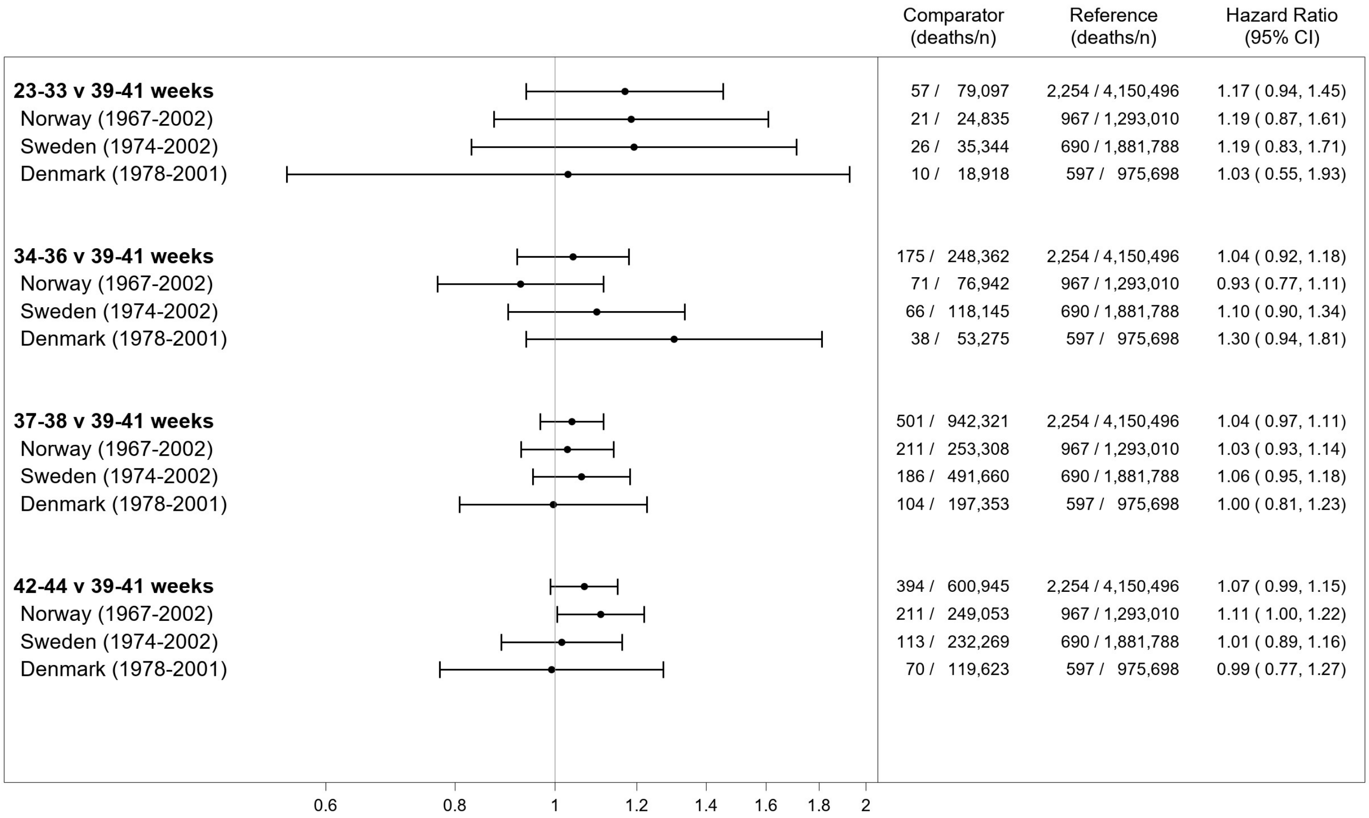

CI: confidence interval, HR: hazard ratio
Cox regression adjusting for birth cohort (<=1985, >1985), sex, birth weight (SD), maternal parity (0,>=1). Cause-specific hazard (Wolders et al. EHJ 2014)
Overall estimate: inverse variance method (Whitehead & Whitehead, Stat Med, 1991). Separately for each comparison, as per Borenstein et al. 2009
Denmark: >=1978 to allow finer categorisation of gestational age; n displayed is the number of individuals included in the analysis, i.e. those with no missing covariate information

© 2021 Risnes K et al. *JAMA Network Open.*

eTable 3. Mortality From Specific NCDs by Country and Overall, Excluding Individuals With Congenital Anomalies, Hazard Ratios (HRs) and 95% CIs by Categories of Gestational Age

	Norway (1967-2002)		Sweden (1974-2002)		Combined	
Gestational age (wks)	deaths/n	HR (95% CI)	deaths/n	HR (95% CI)	deaths/n	HR (95% CI)
Diabetes						
23-36	25 / 98,400	1.87 (1.23, 2.84)	14 / 143,500	2.01 (1.14, 3.53)	39 / 241,900	1.90 (1.36, 2.66)
37-38	43 / 245,893	1.28 (0.92, 1.79)	46 / 470,572	2.06 (1.45, 2.95)	89 / 716,465	1.59 (1.25, 2.02)
39-41	184 / 1,262,188	Reference	90 / 1,810,876	Reference	274 / 3,073,064	Reference
42-44	45 / 242,663	1.11 (0.80, 1.54)	13 / 222,539	0.98 (0.54, 1.76)	58 / 465,202	1.10 (0.82, 1.46)
Chronic lung diseases						
23-36	10 / 98,400	2.27 (1.16, 4.42)	6 / 143,500	2.53 (1.05, 6.10)	16 / 241,900	2.32 (1.37, 3.95)
37-38	12 / 245,893	1.06 (0.57, 1.96)	9 / 470,572	1.26 (0.60, 2.65)	21 / 716,465	1.13 (0.70, 1.82)
39-41	65 / 1,262,188	Reference	30 / 1,810,876	Reference	95 / 3,073,064	Reference
42-44	11 / 242,663	0.75 (0.40, 1.44)	2 / 222,539	0.41 (0.10, 1.74)	13 / 465,202	0.67 (0.37, 1.20)
Stroke						
23-36	18 / 98,400	1.36 (0.84, 2.20)	6 / 143,500	0.74 (0.32, 1.68)	24 / 241,900	1.13 (0.74, 1.71)
37-38	25 / 245,893	0.74 (0.49, 1.12)	19 / 470,572	0.79 (0.48, 1.28)	44 / 716,465	0.76 (0.56, 1.05)
39-41	194 / 1,262,188	Reference	101 / 1,810,876	Reference	295 / 3,073,064	Reference
42-44	42 / 242,663	0.98 (0.70, 1.37)	18 / 222,539	1.10 (0.66, 1.83)	60 / 465,202	1.00 (0.76, 1.33)
Other CVD						
23-36	73 / 98,400	1.37 (1.08, 1.74)	67 / 143,500	1.67 (1.30, 2.16)	140 / 241,900	1.49 (1.25, 1.77)
37-38	176 / 245,893	1.30 (1.10, 1.53)	146 / 470,572	1.14 (0.95, 1.36)	322 / 716,465	1.22 (1.08, 1.38)
39-41	743 / 1,262,188	Reference	528 / 1,810,876	Reference	1,271 / 3,073,064	Reference
42-44	165 / 242,663	1.07 (0.90, 1.27)	90 / 222,539	1.14 (0.91, 1.43)	255 / 465,202	1.09 (0.95, 1.25)

© 2021 Risnes K et al. *JAMA Network Open.*

eFigure 3. All-Cause Mortality by Country and Overall, Excluding Individuals With Congenital Anomalies, Hazard Ratios (HRs) and 95% CIs by Categories of Gestational Age

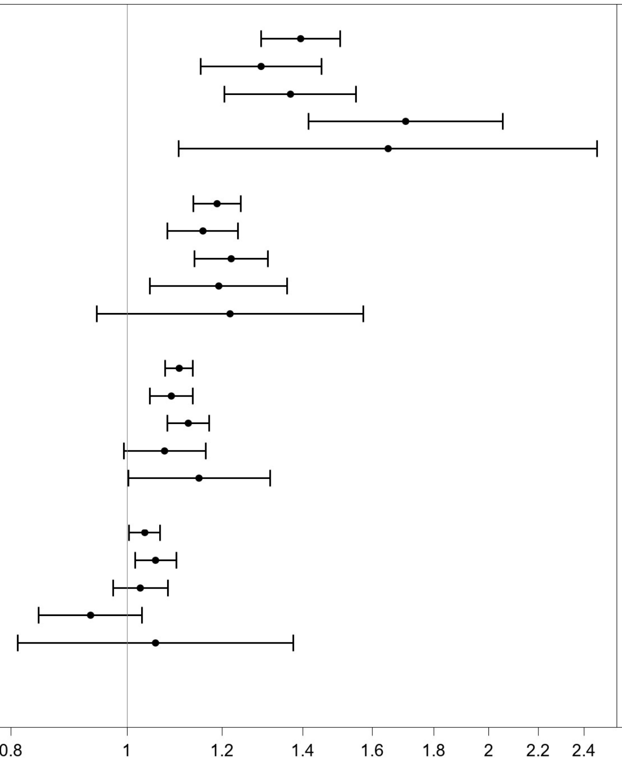

	Comparator (deaths/n)	Reference (deaths/n)	Hazard Ratio (95% CI)
**23-33 v 39-41 weeks**	682 / 76,267	29,874 / 4,202,680	1.39 (1.29, 1.50)
Norway (1967-2002)	292 / 23,930	13,972 / 1,262,188	1.29 (1.15, 1.45)
Sweden (1974-2002)	249 / 31,842	11,067 / 1,810,876	1.37 (1.21, 1.55)
Denmark (1978-2001)	116 / 18,035	3,875 / 961,689	1.71 (1.42, 2.05)
Finland (1987-1990)	25 / 2,460	960 / 167,927	1.65 (1.10, 2.46)
**34-36 v 39-41 weeks**	2,030 / 246,431	29,874 / 4,202,680	1.19 (1.14, 1.24)
Norway (1967-2002)	894 / 74,470	13,972 / 1,262,188	1.16 (1.08, 1.24)
Sweden (1974-2002)	835 / 111,658	11,067 / 1,810,876	1.22 (1.14, 1.31)
Denmark (1978-2001)	237 / 51,555	3,875 / 961,689	1.19 (1.04, 1.36)
Finland (1987-1990)	64 / 8,748	960 / 167,927	1.22 (0.94, 1.57)
**37-38 v 39-41 weeks**	6,871 / 950,258	29,874 / 4,202,680	1.10 (1.08, 1.13)
Norway (1967-2002)	2,772 / 245,893	13,972 / 1,262,188	1.09 (1.04, 1.13)
Sweden (1974-2002)	3,084 / 470,572	11,067 / 1,810,876	1.12 (1.08, 1.17)
Denmark (1978-2001)	748 / 193,018	3,875 / 961,689	1.07 (0.99, 1.16)
Finland (1987-1990)	267 / 40,775	960 / 167,927	1.15 (1.00, 1.31)
**42-44 v 39-41 weeks**	5,257 / 592,631	29,874 / 4,202,680	1.03 (1.00, 1.07)
Norway (1967-2002)	3,105 / 242,663	13,972 / 1,262,188	1.06 (1.01, 1.10)
Sweden (1974-2002)	1,650 / 222,539	11,067 / 1,810,876	1.03 (0.97, 1.08)
Denmark (1978-2001)	443 / 117,836	3,875 / 961,689	0.93 (0.84, 1.03)
Finland (1987-1990)	59 / 9,593	960 / 167,927	1.06 (0.81, 1.38)

CI: confidence interval, HR: hazard ratio *Sensitivity analysis: excluding individuals with congenital anomalies (Norway, Sweden and Denmark only)
Cox regression adjusting for birth cohort (<=1985, >1985), sex, birth weight (SD), maternal parity (0,>=1). Cause-specific hazard (Wolders et al. EHJ 2014)
Overall estimate: inverse variance method (Whitehead & Whitehead, Stat Med, 1991). Separately for each comparison, as per Borenstein et al. 2009
Denmark: >=1978 to allow finer categorisation of gestational age; n displayed is the number of individuals included in the analysis, i.e. those with no missing covariate information

© 2021 Risnes K et al. *JAMA Network Open.*

eFigure 4. All-Cause Mortality by Country and Overall, Restricting Birth Year to 1974 and Onward, Hazard Ratios (HRs) and 95% CIs by Categories of Gestational Age

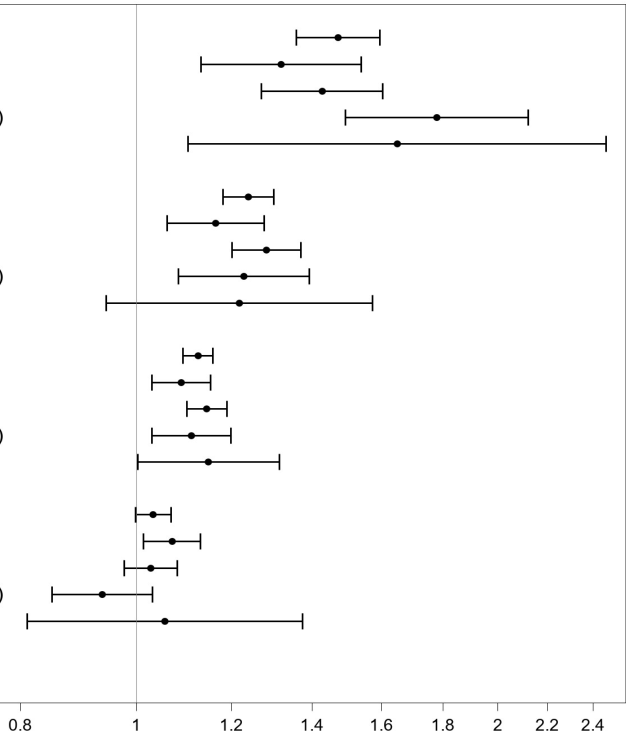

	Comparator (deaths/n)	Reference (deaths/n)	Hazard Ratio (95% CI)
**23-33 v 39-41 weeks**	619 / 77,260	24,017 / 4,024,282	1.47 (1.36, 1.59)
Norway (1974-2002)	168 / 20,538	7,240 / 998,869	1.32 (1.13, 1.54)
Sweden (1974-2002)	295 / 35,344	11,773 / 1,881,788	1.43 (1.27, 1.60)
Denmark (1978-2001)	131 / 18,918	4,044 / 975,698	1.78 (1.49, 2.12)
Finland (1987-1990)	25 / 2,460	960 / 167,927	1.65 (1.10, 2.46)
**34-36 v 39-41 weeks**	1,748 / 241,032	24,017 / 4,024,282	1.24 (1.18, 1.30)
Norway (1974-2002)	474 / 60,864	7,240 / 998,869	1.16 (1.06, 1.28)
Sweden (1974-2002)	949 / 118,145	11,773 / 1,881,788	1.28 (1.20, 1.37)
Denmark (1978-2001)	261 / 53,275	4,044 / 975,698	1.23 (1.08, 1.39)
Finland (1987-1990)	64 / 8,748	960 / 167,927	1.22 (0.94, 1.57)
**37-38 v 39-41 weeks**	5,893 / 931,036	24,017 / 4,024,282	1.13 (1.09, 1.16)
Norway (1974-2002)	1,461 / 201,248	7,240 / 998,869	1.09 (1.03, 1.15)
Sweden (1974-2002)	3,351 / 491,660	11,773 / 1,881,788	1.14 (1.10, 1.19)
Denmark (1978-2001)	814 / 197,353	4,044 / 975,698	1.11 (1.03, 1.20)
Finland (1987-1990)	267 / 40,775	960 / 167,927	1.15 (1.00, 1.31)
**42-44 v 39-41 weeks**	3,935 / 550,251	24,017 / 4,024,282	1.03 (1.00, 1.07)
Norway (1974-2002)	1,639 / 188,766	7,240 / 998,869	1.07 (1.01, 1.13)
Sweden (1974-2002)	1,770 / 232,269	11,773 / 1,881,788	1.03 (0.98, 1.08)
Denmark (1978-2001)	467 / 119,623	4,044 / 975,698	0.94 (0.85, 1.03)
Finland (1987-1990)	59 / 9,593	960 / 167,927	1.06 (0.81, 1.38)

CI: confidence interval, HR: hazard ratio. *Sensitivity analysis: Norway birth year from 1974
Cox regression adjusting for birth cohort (<=1985, >1985), sex, birth weight (SD), maternal parity (0,>=1). Cause-specific hazard (Wolders et al. EHJ 2014)
Overall estimate: inverse variance method (Whitehead & Whitehead, Stat Med, 1991). Separately for each comparison, as per Borenstein et al. 2009
Denmark: >=1978 to allow finer categorisation of gestational age; n displayed is the number of individuals included in the analysis, i.e. those with no missing covariate information

© 2021 Risnes K et al. *JAMA Network Open.*

eFigure 5. All-Cause Mortality by Country and Overall, Restricting Birth Year to 1978 and Onwards, Hazard Ratios (HRs) and 95% CIs by Categories of Gestational Age

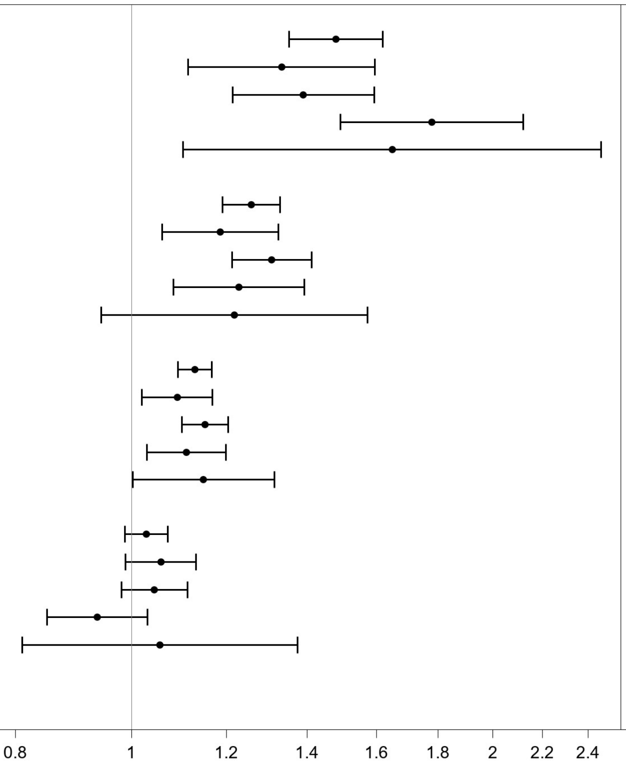

	Comparator (deaths/n)	Reference (deaths/n)	Hazard Ratio (95% CI)
**23-33 v 39-41 weeks**	497 / 71,726	18,148 / 3,618,787	1.48 (1.35, 1.62)
Norway (1978-2002)	123 / 18,509	4,855 / 857,090	1.33 (1.11, 1.60)
Sweden (1978-2002)	218 / 31,839	8,289 / 1,618,072	1.39 (1.21, 1.59)
Denmark (1978-2001)	131 / 18,918	4,044 / 975,698	1.78 (1.49, 2.12)
Finland (1987-1990)	25 / 2,460	960 / 167,927	1.65 (1.10, 2.46)
**34-36 v 39-41 weeks**	1,373 / 219,560	18,148 / 3,618,787	1.26 (1.19, 1.33)
Norway (1978-2002)	333 / 53,463	4,855 / 857,090	1.19 (1.06, 1.32)
Sweden (1978-2002)	715 / 104,074	8,289 / 1,618,072	1.31 (1.21, 1.41)
Denmark (1978-2001)	261 / 53,275	4,044 / 975,698	1.23 (1.08, 1.39)
Finland (1987-1990)	64 / 8,748	960 / 167,927	1.22 (0.94, 1.57)
**37-38 v 39-41 weeks**	4,634 / 853,368	18,148 / 3,618,787	1.13 (1.09, 1.17)
Norway (1978-2002)	1,013 / 177,310	4,855 / 857,090	1.09 (1.02, 1.17)
Sweden (1978-2002)	2,540 / 437,930	8,289 / 1,618,072	1.15 (1.10, 1.20)
Denmark (1978-2001)	814 / 197,353	4,044 / 975,698	1.11 (1.03, 1.20)
Finland (1987-1990)	267 / 40,775	960 / 167,927	1.15 (1.00, 1.31)
**42-44 v 39-41 weeks**	2,692 / 470,784	18,148 / 3,618,787	1.03 (0.99, 1.07)
Norway (1978-2002)	1,068 / 158,581	4,855 / 857,090	1.06 (0.99, 1.13)
Sweden (1978-2002)	1,098 / 182,987	8,289 / 1,618,072	1.04 (0.98, 1.11)
Denmark (1978-2001)	467 / 119,623	4,044 / 975,698	0.94 (0.85, 1.03)
Finland (1987-1990)	59 / 9,593	960 / 167,927	1.06 (0.81, 1.38)

CI: confidence interval, HR: hazard ratio. *Sensitivity analysis: Norway and Sweden birth year from 1978
Cox regression adjusting for birth cohort (<=1985, >1985), sex, birth weight (SD), maternal parity (0,>=1). Cause-specific hazard (Wolders et al. EHJ 2014)
Overall estimate: inverse variance method (Whitehead & Whitehead, Stat Med, 1991). Separately for each comparison, as per Borenstein et al. 2009
Denmark: >=1978 to allow finer categorisation of gestational age; n displayed is the number of individuals included in the analysis, i.e. those with no missing covariate information

© 2021 Risnes K et al. *JAMA Network Open.*

eFigure 6. Noncommunicable Disease Mortality by Country and Overall, Restricting Birth Year to 1974 and Onward, Hazard Ratios (HRs) and 95% CIs by Categories of Gestational Age

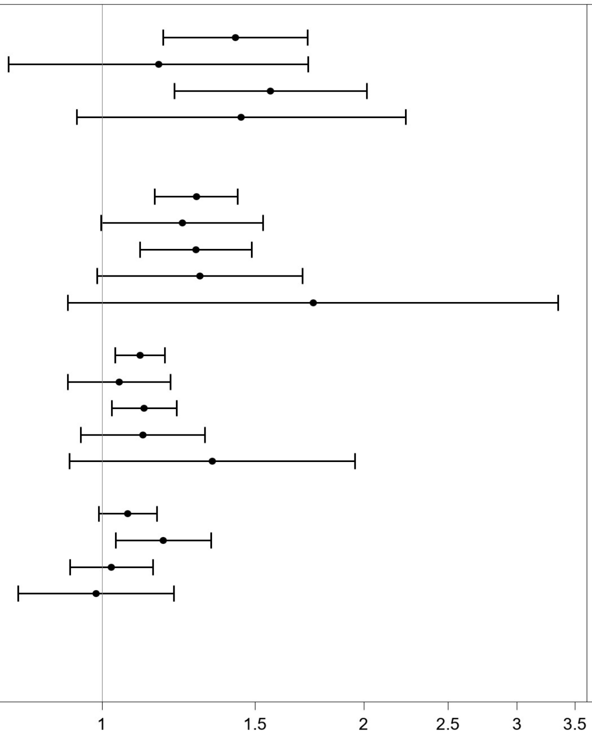

	Comparator (deaths/n)	Reference (deaths/n)	Hazard Ratio (95% CI)
**23-33 v 39-41 weeks**	108 / 77,260	4,735 / 4,024,282	1.42 (1.18, 1.72)
Norway (1974-2002)	25 / 20,538	1,309 / 998,869	1.16 (0.78, 1.73)
Sweden (1974-2002)	61 / 35,344	2,451 / 1,881,788	1.56 (1.21, 2.02)
Denmark (1978-2001)	21 / 18,918	867 / 975,698	1.45 (0.94, 2.23)
Finland (1987-1990)	Deaths <=5		
**34-36 v 39-41 weeks**	345 / 241,032	4,735 / 4,024,282	1.28 (1.15, 1.43)
Norway (1974-2002)	90 / 60,864	1,309 / 998,869	1.24 (1.00, 1.53)
Sweden (1974-2002)	189 / 118,145	2,451 / 1,881,788	1.28 (1.11, 1.49)
Denmark (1978-2001)	56 / 53,275	867 / 975,698	1.30 (0.99, 1.70)
Finland (1987-1990)	10 / 8,748	108 / 167,927	1.75 (0.91, 3.35)
**37-38 v 39-41 weeks**	1,116 / 931,036	4,735 / 4,024,282	1.11 (1.04, 1.18)
Norway (1974-2002)	250 / 201,248	1,309 / 998,869	1.05 (0.91, 1.20)
Sweden (1974-2002)	660 / 491,660	2,451 / 1,881,788	1.12 (1.03, 1.22)
Denmark (1978-2001)	170 / 197,353	867 / 975,698	1.11 (0.95, 1.31)
Finland (1987-1990)	36 / 40,775	108 / 167,927	1.34 (0.92, 1.95)
**42-44 v 39-41 weeks**	795 / 550,251	4,735 / 4,024,282	1.07 (0.99, 1.16)
Norway (1974-2002)	310 / 188,766	1,309 / 998,869	1.18 (1.04, 1.33)
Sweden (1974-2002)	378 / 232,269	2,451 / 1,881,788	1.03 (0.92, 1.14)
Denmark (1978-2001)	102 / 119,623	867 / 975,698	0.98 (0.80, 1.21)
Finland (1987-1990)	Deaths <=5		

CI: confidence interval, HR: hazard ratio. *Sensitivity analysis: Norway birth year from 1974
Cox regression adjusting for birth cohort (<=1985, >1985), sex, birth weight (SD), maternal parity (0,>=1). Cause-specific hazard (Wolders et al. EHJ 2014)
Overall estimate: inverse variance method (Whitehead & Whitehead, Stat Med, 1991). Separately for each comparison, as per Borenstein et al. 2009
Denmark: >=1978 to allow finer categorisation of gestational age; n displayed is the number of individuals included in the analysis, i.e. those with no missing covariate information

© 2021 Risnes K et al. *JAMA Network Open.*

eFigure 7. Noncommunicable Disease Mortality by Country and Overall, Restricting Birth Year to 1978 and Onwards, Hazard Ratios (HRs) and 95% CIs by Categories of Gestational Age

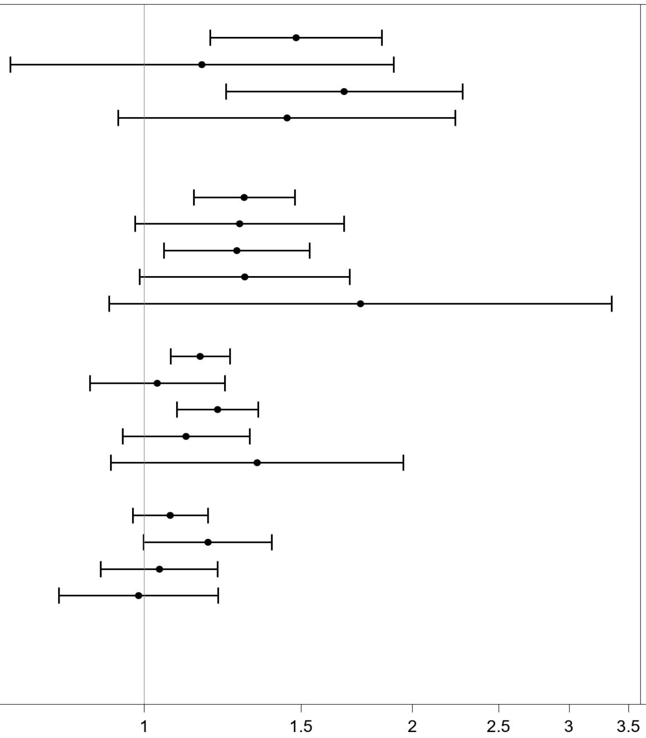

	Comparator (deaths/n)	Reference (deaths/n)	Hazard Ratio (95% CI)
**23-33 v 39-41 weeks**	81 / 71,726	3,195 / 3,618,787	1.48 (1.19, 1.85)
Norway (1978-2002)	16 / 18,509	773 / 857,090	1.16 (0.71, 1.91)
Sweden (1978-2002)	43 / 31,839	1,447 / 1,618,072	1.68 (1.24, 2.28)
Denmark (1978-2001)	21 / 18,918	867 / 975,698	1.45 (0.94, 2.23)
Finland (1987-1990)	Deaths <=5		
**34-36 v 39-41 weeks**	241 / 219,560	3,195 / 3,618,787	1.30 (1.14, 1.48)
Norway (1978-2002)	57 / 53,463	773 / 857,090	1.28 (0.98, 1.68)
Sweden (1978-2002)	118 / 104,074	1,447 / 1,618,072	1.27 (1.05, 1.53)
Denmark (1978-2001)	56 / 53,275	867 / 975,698	1.30 (0.99, 1.70)
Finland (1987-1990)	10 / 8,748	108 / 167,927	1.75 (0.91, 3.35)
**37-38 v 39-41 weeks**	817 / 853,368	3,195 / 3,618,787	1.16 (1.07, 1.25)
Norway (1978-2002)	152 / 177,310	773 / 857,090	1.03 (0.87, 1.23)
Sweden (1978-2002)	459 / 437,930	1,447 / 1,618,072	1.21 (1.09, 1.34)
Denmark (1978-2001)	170 / 197,353	867 / 975,698	1.11 (0.95, 1.31)
Finland (1987-1990)	36 / 40,775	108 / 167,927	1.34 (0.92, 1.95)
**42-44 v 39-41 weeks**	478 / 470,784	3,195 / 3,618,787	1.07 (0.97, 1.18)
Norway (1978-2002)	178 / 158,581	773 / 857,090	1.18 (1.00, 1.39)
Sweden (1978-2002)	193 / 182,987	1,447 / 1,618,072	1.04 (0.89, 1.21)
Denmark (1978-2001)	102 / 119,623	867 / 975,698	0.98 (0.80, 1.21)
Finland (1987-1990)	Deaths <=5		

CI: confidence interval, HR: hazard ratio. *Sensitivity analysis: Norway and Sweden birth year from 1978
Cox regression adjusting for birth cohort (<=1985, >1985), sex, birth weight (SD), maternal parity (0,>=1). Cause-specific hazard (Wolders et al. EHJ 2014)
Overall estimate: inverse variance method (Whitehead & Whitehead, Stat Med, 1991). Separately for each comparison, as per Borenstein et al. 2009
Denmark: >=1978 to allow finer categorisation of gestational age; n displayed is the number of individuals included in the analysis, i.e. those with no missing covariate information

© 2021 Risnes K et al. *JAMA Network Open.*

eFigure 8. Cardiovascular Disease Mortality by Country and Overall, Restricting Birth Year to 1974 and Onward, Hazard Ratios (HRs) and 95% CIs by Categories of Gestational Age

	Comparator (deaths/n)	Reference (deaths/n)	Hazard Ratio (95% CI)
**23-33 v 39-41 weeks**	46 / 74,800	1,225 / 3,856,355	2.22 (1.65, 2.98)
Norway (1974-2002)	11 / 20,538	315 / 998,869	2.00 (1.09, 3.65)
Sweden (1974-2002)	26 / 35,344	690 / 1,881,788	2.26 (1.52, 3.35)
Denmark (1978-2001)	9 / 18,918	220 / 975,698	2.38 (1.21, 4.66)
**34-36 v 39-41 weeks**	114 / 232,284	1,225 / 3,856,355	1.60 (1.32, 1.94)
Norway (1974-2002)	31 / 60,864	315 / 998,869	1.77 (1.22, 2.56)
Sweden (1974-2002)	66 / 118,145	690 / 1,881,788	1.55 (1.20, 1.99)
Denmark (1978-2001)	17 / 53,275	220 / 975,698	1.51 (0.92, 2.48)
**37-38 v 39-41 weeks**	300 / 890,261	1,225 / 3,856,355	1.14 (1.00, 1.29)
Norway (1974-2002)	69 / 201,248	315 / 998,869	1.21 (0.93, 1.57)
Sweden (1974-2002)	186 / 491,660	690 / 1,881,788	1.11 (0.94, 1.31)
Denmark (1978-2001)	45 / 197,353	220 / 975,698	1.15 (0.84, 1.59)
**42-44 v 39-41 weeks**	212 / 540,658	1,225 / 3,856,355	1.08 (0.93, 1.25)
Norway (1974-2002)	72 / 188,766	315 / 998,869	1.08 (0.83, 1.40)
Sweden (1974-2002)	113 / 232,269	690 / 1,881,788	1.09 (0.89, 1.33)
Denmark (1978-2001)	27 / 119,623	220 / 975,698	1.02 (0.68, 1.52)

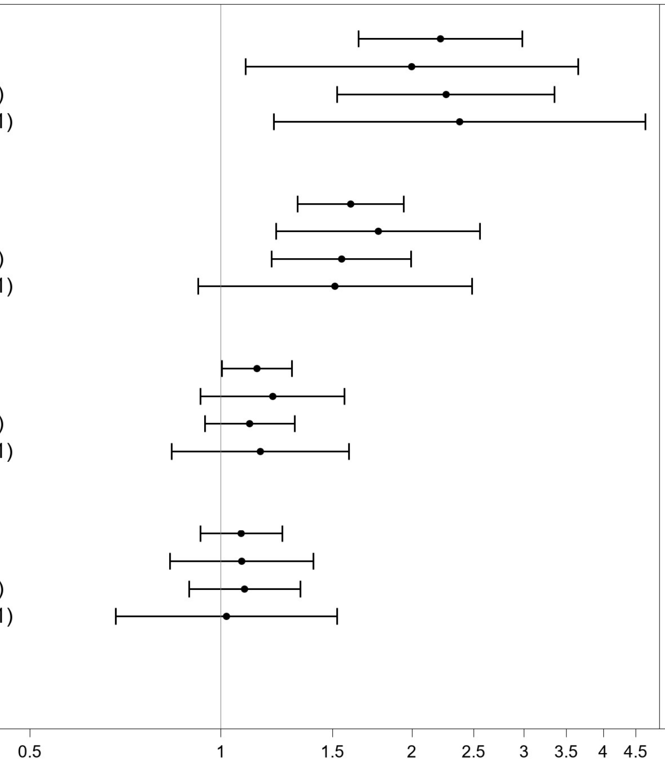

CI: confidence interval, HR: hazard ratio. *Sensitivity analysis: Norway birth year from 1974
Cox regression adjusting for birth cohort (<=1985, >1985), sex, birth weight (SD), maternal parity (0,>=1). Cause-specific hazard (Wolders et al. EHJ 2014)
Overall estimate: inverse variance method (Whitehead & Whitehead, Stat Med, 1991). Separately for each comparison, as per Borenstein et al. 2009
Denmark: >=1978 to allow finer categorisation of gestational age; n displayed is the number of individuals included in the analysis, i.e. those with no missing covariate information

© 2021 Risnes K et al. *JAMA Network Open.*

eFigure 9. Cardiovascular Disease Mortality by Country and Overall, Restricting Birth Year to 1978 and Onwards, Hazard Ratios (HRs) and 95% CIs by Categories of Gestational Age

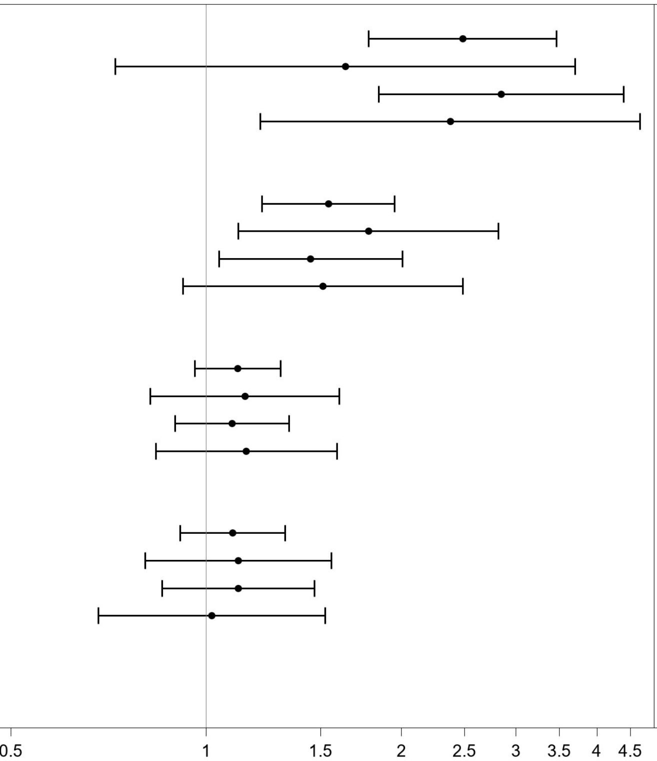

	Comparator (deaths/n)	Reference (deaths/n)	Hazard Ratio (95% CI)
**23-33 v 39-41 weeks**	37 / 69,266	834 / 3,450,860	2.48 (1.78, 3.47)
Norway (1978-2002)	6 / 18,509	192 / 857,090	1.64 (0.72, 3.70)
Sweden (1978-2002)	22 / 31,839	422 / 1,618,072	2.85 (1.85, 4.39)
Denmark (1978-2001)	9 / 18,918	220 / 975,698	2.38 (1.21, 4.66)
**34-36 v 39-41 weeks**	77 / 210,812	834 / 3,450,860	1.54 (1.22, 1.95)
Norway (1978-2002)	20 / 53,463	192 / 857,090	1.78 (1.12, 2.82)
Sweden (1978-2002)	40 / 104,074	422 / 1,618,072	1.45 (1.05, 2.01)
Denmark (1978-2001)	17 / 53,275	220 / 975,698	1.51 (0.92, 2.48)
**37-38 v 39-41 weeks**	209 / 812,593	834 / 3,450,860	1.12 (0.96, 1.30)
Norway (1978-2002)	42 / 177,310	192 / 857,090	1.15 (0.82, 1.60)
Sweden (1978-2002)	122 / 437,930	422 / 1,618,072	1.10 (0.90, 1.34)
Denmark (1978-2001)	45 / 197,353	220 / 975,698	1.15 (0.84, 1.59)
**42-44 v 39-41 weeks**	133 / 461,191	834 / 3,450,860	1.10 (0.91, 1.32)
Norway (1978-2002)	45 / 158,581	192 / 857,090	1.12 (0.81, 1.56)
Sweden (1978-2002)	61 / 182,987	422 / 1,618,072	1.12 (0.85, 1.47)
Denmark (1978-2001)	27 / 119,623	220 / 975,698	1.02 (0.68, 1.52)

CI: confidence interval, HR: hazard ratio. *Sensitivity analysis: Norway and Sweden birth year from 1978
Cox regression adjusting for birth cohort (<=1985, >1985), sex, birth weight (SD), maternal parity (0,>=1). Cause-specific hazard (Wolders et al. EHJ 2014)
Overall estimate: inverse variance method (Whitehead & Whitehead, Stat Med, 1991). Separately for each comparison, as per Borenstein et al. 2009
Denmark: >=1978 to allow finer categorisation of gestational age; n displayed is the number of individuals included in the analysis, i.e. those with no missing covariate information

© 2021 Risnes K et al. *JAMA Network Open.*

*eTable 4. Hazard Ratio (95% CI) for Sibling Analysis**

Mortality Outcome	Gestational age (wks)	Deaths / N	HR (95% CI)
All-causes	23-33 weeks	460 / 50,782	1.34 (1.15, 1.57)
	34-36 weeks	1,516 / 167,010	1.18 (1.09, 1.29)
	37-38 weeks	4,923 / 645,395	1.08 (1.03, 1.14)
	39-41 weeks	21,076 / 2,761,361	Reference
	42-44 weeks	4,040 / 412,059	1.03 (0.98, 1.09)
NCDs	23-33 weeks	101 / 50,782	1.29 (0.90, 1.84)
	34-36 weeks	310 / 167,010	1.15 (0.95, 1.40)
	37-38 weeks	1,029 / 645,395	1.07 (0.96, 1.20)
	39-41 weeks	4,652 / 2,761,361	Reference
	42-44 weeks	914 / 412,059	1.04 (0.92, 1.17)
Cardiovasular disease	23-33 weeks	38 / 50,782	1.75 (0.91, 3.34)
	34-36 weeks	103 / 167,010	1.06 (0.75, 1.52)
	37-38 weeks	297 / 645,395	1.10 (0.89, 1.37)
	39-41 weeks	1,288 / 2,761,361	Reference
	42-44 weeks	256 / 412,059	1.01 (0.81, 1.27)

**Siblings analysis population includes any individual with siblings. NCD: Non-communicable diseases. Stratified Cox regression including mothers as strata, adjusting for country, birth cohort (<=1985, >1985), sex, birth weight (SD), maternal parity (0,>=1). Cause-specific hazard (Wolders et al. EHJ 2014)*

© 2021 Risnes K et al. *JAMA Network Open.*
